# A novel role for *gag* as a *cis*-acting element regulating RNA structure, dimerization and packaging in HIV-1 lentiviral vectors

**DOI:** 10.1093/nar/gkab1206

**Published:** 2021-12-20

**Authors:** Eirini Vamva, Alex Griffiths, Conrad A Vink, Andrew M L Lever, Julia C Kenyon

**Affiliations:** University of Cambridge Department of Medicine, Cambridge Biomedical Campus, Cambridge CB2 0QQ, UK; GlaxoSmithKline, Gunnels Wood Road, Stevenage SG1 2NY, UK; University of Cambridge Department of Medicine, Cambridge Biomedical Campus, Cambridge CB2 0QQ, UK; GlaxoSmithKline, Gunnels Wood Road, Stevenage SG1 2NY, UK; University of Cambridge Department of Medicine, Cambridge Biomedical Campus, Cambridge CB2 0QQ, UK; Department of Medicine, Yong Loo Lin School of Medicine 119228, Singapore; University of Cambridge Department of Medicine, Cambridge Biomedical Campus, Cambridge CB2 0QQ, UK; Department of Microbiology and Immunology, Yong Loo Lin School of Medicine, 117545, Singapore; Homerton College, Hills Road, Cambridge CB2 8PH, UK

## Abstract

Clinical usage of lentiviral vectors is now established and increasing but remains constrained by vector titer with RNA packaging being a limiting factor. Lentiviral vector RNA is packaged through specific recognition of the packaging signal on the RNA by the viral structural protein Gag. We investigated structurally informed modifications of the 5′ leader and *gag* RNA sequences in which the extended packaging signal lies, to attempt to enhance the packaging process by facilitating vector RNA dimerization, a process closely linked to packaging. We used in-gel SHAPE to study the structures of these mutants in an attempt to derive structure-function correlations that could inform optimized vector RNA design. In-gel SHAPE of both dimeric and monomeric species of RNA revealed a previously unreported direct interaction between the U5 region of the HIV-1 leader and the downstream *gag* sequences. Our data suggest a structural equilibrium exists in the dimeric viral RNA between a metastable structure that includes a U5–*gag* interaction and a more stable structure with a U5–AUG duplex. Our data provide clarification for the previously unexplained requirement for the 5′ region of *gag* in enhancing genomic RNA packaging and provide a basis for design of optimized HIV-1 based vectors.

## INTRODUCTION

**Figure 1.**
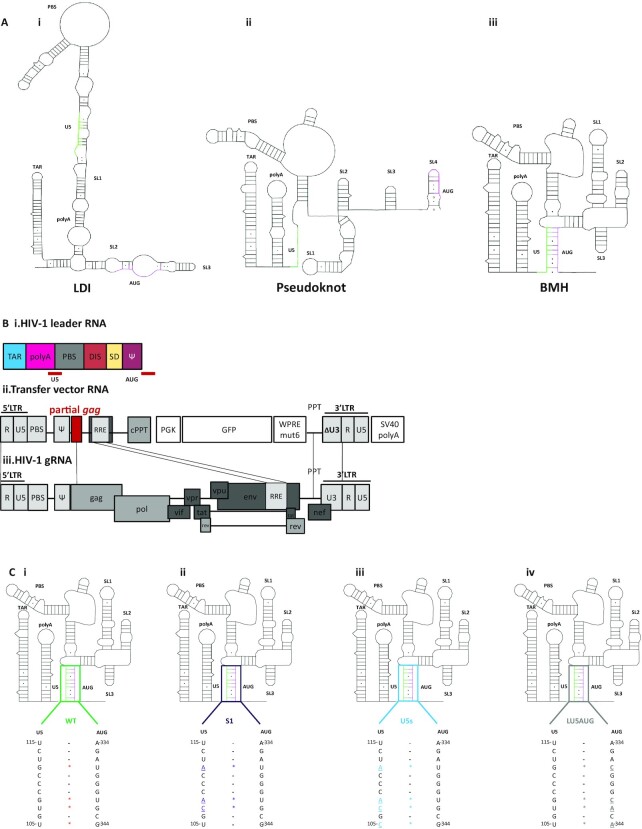
Mutational design. (**A**) Schematics of established structural conformations of the HIV-1 leader RNA with parts of the U5 and *gag* regions that engage in the established U5–AUG duplex coloured in green (U5) and purple (*gag*). (i) LDI (long distance interaction) structure. (ii) Pseudoknot structure. (iii) BMH (branched multiple hairpin) structure. (**B**, i) The *cis*-acting elements of the HIV-1 leader with the sequences of the U5 region that interact with the *gag* sequences around the start codon are indicated. (ii) elements included in the third generation HIV-1 derived SIN lentiviral transfer vector expressed RNA. (iii) The corresponding elements shown on the HXB2-HIV-1 gRNA. (**C**) Schematic diagram of the BMH structure and the nucleotide positions participating in the formation of the U5–AUG and base pairing of the WT and mutant U5–AUG duplexes. Noncanonical G–U pairing in the WT U5–AUG is indicated in red. Mutated nucleotides are indicated in **bold** and in colour and are underlined. (i) WT U5–AUG interaction. (ii) S1 mutant containing three point mutations in the U5 region that increase the stability of U5–AUG by four Watson–Crick bonds. (iii) U5s mutant containing four point mutations in the U5 region that increase the stability of U5–AUG by six Watson–Crick bonds. (iv) LU5AUG mutant containing four point mutations in the *gag* region around the AUG that increase the stability of U5–AUG by six Watson–Crick bonds.

HIV-1 derived lentiviral vectors (LVs) are increasingly being used in clinical applications due to their safe integration pattern (1), sustainable gene expression (2) and mitosis independent cellular transduction (3, 4). Retention of the minimal essential regions of HIV-1 to maximize vector transgene capacity while optimizing delivery and obviating recombination risks have been central to development of clinically useful vectors (5). The 5′-leader, the most conserved region (http://www.hiv.lanl.gov/), has proven indispensable as it contains the *cis*-acting elements that regulate a variety of essential processes including transcriptional activation, reverse transcription, translation, splicing, genome dimerization and packaging (6). It is proposed to adopt different conformations favouring either translation or genome dimerization and encapsidation between which it switches in response to as yet poorly understood signals (7–11) (Figure 1A). Structure(s) of the leader and of subregions including the packaging signal (*psi* – Ψ) regions have been derived by a number of groups using a diversity of techniques (6, 8, 10–14). The range of published putative structures is explained by the intrinsic flexibility and structural instability of the region, which is required for it to adopt different functional roles (Figure 1A). Studying the conformers the region can form individually has been technically challenging, and attempts to visualize multiple separate HIV-1 structures in cells or virions have not worked, likely due to the presence of so many conformers within a biological sample. We previously developed in-gel SHAPE (selective 2′OH acylation analysed by primer extension), which allows for the structural probing of individual conformers within a mixed population, *in vitro* (10). The use of this technique allowed us to show conclusively that a structural switch occurred between monomeric and dimeric RNA. RNA dimerization is as yet incompletely understood but is believed to be initiated by a ‘kissing loop’ (15) intermolecular interaction at a palindromic sequence in the dimer initiation site (DIS) (16). In the unspliced monomeric viral RNA, the DIS loop interacts with the U5 region so as to form a lower free energy structure which is easier for the ribosomes to scan and has the AUG start codon of *gag* more accessible (pseudoknot, Figure 1A) (8, 12). A conformational switch leaves the DIS exposed and occludes the *gag* start codon by pairing it with a region in the U5 sequence to form the U5–AUG structural motif [BMH (branched multiple hairpin model), Figure 1A]. This makes the same RNA template dimerization competent (13, 17, 18). Whether the Gag protein binds to the monomer and triggers this structural switch to promote dimerization, or whether the monomer dimerizes in its absence and then recruits Gag, is a subject of debate. However, there is a consensus that the dimerized, Gag-bound mature dimeric structure contains the U5–AUG motif. Whilst much is known about the structure-function aspects of the HIV leader RNA it has been clear that sequences within the proximal Gag coding region also play a role in gRNA packaging (19–23). Whether this is structure or sequence dependent, or both, is unknown, however 3^rd^ generation advanced lentiviral transfer vector systems (Figure 1B) routinely include the first 361 bp of the *gag* coding region since it has been shown empirically to enhance their efficiency (24). Omission of this region showed that it was essential in *cis* for efficient vector propagation (25), and RNA packaging (19).

In this study we investigated the optimization of the dimerization properties of HIV-1 derived lentiviral vector RNAs and the effect on packaging, through structure informed genome modifications targeting the U5–AUG interaction. This failed to reveal a simple relationship between modulation of dimerization, packaging and infectivity. As anticipated, U5–AUG stabilized mutants were characterized by an increased propensity to dimerize. Despite this and the established relationship between dimerization and packaging (26), these mutations did not increase packaging and actually decreased infectivity; important findings that add to the ongoing debate on the link between these processes (27–32). We therefore extended our analysis to include the 5′*gag* region to attempt to elucidate the functional roles of these additional RNA sequences seeking to relate this to the known inter and intramolecular interactions in the leader RNA.

In-gel SHAPE was employed to study the influence of the *gag* region on the structure of the monomeric and dimeric vector RNA. SHAPE identifies RNA backbone flexibility, an indication of whether nucleotides are base-paired or not, and is used in combination with minimal free energy prediction, to generate structural models (33). In-gel SHAPE was specifically chosen as it enables us to separate and visualize the individual structures adopted by the RNA. Whilst there can be discrepancies between structures found *in vivo* and *in vitro*, the *in vivo* situation carries with it the additional complications of such a structurally flexible RNA as this being found in a variety structures, and of not knowing when and where proteins and other RNAs are bound, affecting the NMIA binding signature and significantly influencing the modelling. We thus chose to use in-gel SHAPE as it enabled us to probe individual structural conformations accurately. At 690 nt for the monomer, the dimeric HIV-1 RNA studied here is close to the maximum size of RNA the technique can be used for, hence it is not currently possible to study these RNAs in-gel in the presence of the Gag protein as the complexes formed are too large to allow their extraction from the gel (34). However, where the mutant RNA was packaged in detectable quantities, the dimerization behaviour of the mutant RNAs was confirmed in virions.

We found that inclusion of 3′ *gag* sequences had no effect on the structure of the monomeric WT vector RNA leader, which remained constant, but their inclusion did have a profound effect on the conformation of the dimeric species leader. In the latter, addition of *gag* sequences downstream of the HIV-1 UTR was accompanied by a decrease in SHAPE reactivity in the packaging signal, suggesting that *gag* had a stabilizing effect on the structure of *psi*. Direct interactions between the polyA (nts 61–95) and U5 (nts 103–122) elements of the HIV-1 leader and *gag* sequences (nts 621–660 and 433–452, respectively) were observed in our structural models. We chose to interrogate the RNA structures present and to derive our structural models in the absence of the Gag protein as the timing of Gag's role in the packaging process is not certain and as this approach enabled us to separate and probe individual RNA structures under native conditions. We then applied further techniques to assess the relevance of our *in vitro*-derived structures to the viral lifecycle. As HIV-1 genetic variation is high, phylogenetic analyses are routinely used to assess whether structures modelled using *in vitro* analyses are evolutionarily conserved (35). Our phylogenetic analyses suggested that this interaction has been highly conserved, strongly implying biological function *in vivo*. To investigate the biological relevance of this structure further, we also assessed the behaviour of wild-type and mutant transfer vectors in cells, the results of which increased support for our proposed structural model.

The presence of U5–*gag* interactions appeared to reduce the stability of the U5–AUG pairing in the dimeric RNA. This points to an expanded equilibrium of interchanging structures that regulate dimerization. Our findings lead to a model in which early formation of the dimer involves novel interactions between the U5 and *gag* sequence that expands the structural ensemble of the RNA and enhances a global conformational change in the viral leader, facilitating formation of a dimeric RNA species and an infectious viral particle. As part of this the involvement of a specific U5 poly-GU bulge region has been identified in the dimerization switch. Our findings help to consolidate disparate observations in the lentiviral packaging field and to explain the functional necessity for extended packaging signal sequences within the *gag* gene.

## MATERIALS AND METHODS

### In-gel SHAPE

#### In vitro production of RNA

A 416 or a 690 nucleotide PCR fragment including the sequences between TAR and the minimal or extended *gag* was amplified by PCR using Phusion DNA polymerase (NEB, # M0530S) from template pCCL-eGFP (Addgene, #10880) or corresponding mutants described below using primers Fw 5′-TAATACGACTCACTATAG GGTCTCTCTGGTTAGACCAGATCTG-3′, Rv_(416nt)_ 5′-CTTTCCCCCTGGCCTTAACC-3′, Rv_(690nt)_ 5′- CTTGCTGTGCGGTGGTCT-3′. PCR products were gel extracted and purified using the NEB Monarch extraction kit (#T1020S). The PCR product was eluted in a 15 μl final volume. *In vitro* transcription was carried out using the MEGAScript T7 transcription kit (Thermo Fisher, #AM1334). Reactions contained extracted PCR product (150 ng), 1x transcription buffer, 10 mM each ATP, TTP, CTP and GTP, and 4 μl of T7 Polymerase enzyme mix in a final volume of 40 μl. The mix was incubated at 37°C for 4h and the product was subsequently treated with 6 U Turbo DNase for 1 h at 37°C. The RNA product was purified by lithium chloride precipitation, resuspended in RNase-free water and stored at −80°C.

#### In-gel chemical acylation of RNAs

45 μg of RNA were resuspended in 160 μl of renaturation buffer (10 mM Tris pH 8, 100 mM KCl and 0.1 mM EDTA) in a microfuge tube, heated at 85°C for 5 min and slow-cooled to room temperature by placing the heat block insert containing the microfuge tube onto the bench. The RNA was subsequently refolded in 40 μl of 5x refolding dimerization buffer [40 mM Tris pH 8, 5 mM magnesium chloride (MgCl_2_), 130 mM KCl and 50 mM sodium cacodylate pH 7.5]. The mixture was incubated at 37°C for 30 min. 60 μl of Orange G native loading dye (1 × TBM (89 mM Tris Base, 89 mM Boric acid and 0.1 mM MgCl_2_), 5% (w/v glycerol, 0.1% (w/v) orange G dye) were added and 20 μl of the treated RNA were loaded in each lane of a 20 cm × 18.5-cm 4% (w/v) acrylamide/bis 19:1 gel prepared with 1× TBM (89 mM Tris base, 89 mM boric acid and 0.1 mM MgCl_2_). Gel electrophoresis was set at 90 V for 1h and 100 V for four additional hours. One fragment corresponding to the RiboRuler Low Range RNA ladder (RbLR ladder) (Thermo Fisher Scientific, #SM1833) and one RNA sample were excised and stained for 10 min with 1.3 μM ethidium bromide in 1× TBM and visualized under UV to detect the position of the dimeric and monomeric RNAs. The ethidium bromide-stained gel fragments were aligned with the remaining unstained gel, from which gel pieces corresponding to the dimeric and monomeric RNA were excised using a scalpel. The expected size of the monomeric RNA containing the *gag* sequences is 690 nt and should be observed between the second and third bands of the RbLR ladder, which correspond to 800 nt and 600 nt, respectively. Similarly the expected size of the dimeric RNA containing the *gag* sequences is 1380 nt and should be observed above the top band of the RbLR ladder which corresponds to 1000 nt. Both the dimeric and monomeric gel pieces were divided into two equal parts, with one fragment incubated in 1× TBM containing 10% (v/v) DMSO, and the other one in 1× TBM containing 10% (v/v) DMSO and 10 mM *N*-methylisatoic anhydride (NMIA). The 4 gel pieces were incubated at room temperature for 1 h and were then washed three times in 1× TAE (40 mM Tris-acetate and 1 mM EDTA pH 8.3). The gel pieces were minced and the RNA electroeluted using the BIO-RAD model 422 Electro-Eluter at 40 mA for 1h 30min. RNA was precipitated with 300 mM sodium acetate pH5.5 and 2.5× volume of ethanol and recovered by centrifugation, washed with 70% ethanol and resuspended in 10 μl of water. RNA concentration was determined by spectrophotometry and gel bands for dimerization analysis were quantified using ImageJ (https://imagej.nih.gov/ij/).

#### Reverse transcription and structural analysis

1000 ng of each RNA sample were each resuspended in 12 μl of 2.1 mM Tris pH 8.0, 42 μM EDTA. 5 nmol of 6FAM™-labelled reverse (Rv) primer (Applied Biosystems) was added to the dimeric and monomeric NMIA-treated samples and 5 nmol of VIC^®^-labelled Rv primer (Applied Biosystems) were added to the dimeric and monomeric negative control samples. Primers were annealed to the RNA at 85°C for 1 min, 60°C for 5 min and 35°C for 5 min. 8 μl of the reverse transcription mix (Invitrogen, #18080044) [100 U of Superscript III reverse transcriptase (RT), 1.5× SSIII RT buffer, 12.5 mM dithiothreitol (DTT), 5M betaine, 1.25 mM deoxycytidine triphosphate (dCTP), deoxyadenosine triphosphate (dATP), deoxyuridine triphosphate (dUTP) and 7-deaza-deoxyguanosine triphosphate (dGTP)] were added to each sample and were incubated at 55°C for 50 min. RNA was degraded with 200 mM NaOH at 95°C for 5 min and cooled on ice for 5 min. The samples were then treated with 200 mM HCl and were precipitated as described above. The cDNA produced was combined with two sequencing ladders produced using the Thermo Sequenase Cycle Sequencing Kit (Applied Biosystems, #785001KT) and the NED™ and PET^®^ labelled Rv primers (Applied Biosystems) according to the manufacturer's instructions. Sequencing ladders were precipitated with 300 mM sodium acetate and 2.5× volume of ethanol and recovered by centrifugation, washed with 70% ethanol and resuspended in 30 μl of water. RNA samples were mixed with the sequencing ladders in a 1:1:1 concentration ratio and were fractionated by fragment analysis in an Applied Biosystems 3730xl analyser. Data were analysed with SHAPEFinder software (36) and normalized as previously described (10). Single level nucleotide structural modelling was performed using the RNAstructure software (https://rna.urmc.rochester.edu/) where RNA secondary structure is predicted using SHAPE data and free energy constraints. Structures were drawn using XRNA (http://rna.ucsc.edu/rnacenter/xrna/).

#### Antisense oligonucleotide targeting of the polyA–gag and the U5–gag interactions

Antisense oligonucleotides_o1, 5′ ATCTTGTCTAAAGCTTCC 3′; and_o2, 5′ GTTCTAGCTCCCTGCTTGCCC 3′; were designed to target the putative polyA–*gag* and U5–*gag* interactions respectively. A control scrambled oligonucleotide sequence 5′ TCGAGTGCCCGAAGGATAGCTA 3′; (37) was also designed. The three oligos were analysed with the alignment search tool nucleotide BLAST (https://blast.ncbi.nlm.nih.gov/Blast.cgi) to ensure that there were no predicted interactions outside of the targeted binding site on the 690 nt WT RNA sequence for o1 and o2, and no predicted interactions for the scrambled oligonucleotide. Oligonucleotides were resuspended in water at a concentration of 10 pmol/μl. 1500 ng of the 690 nt WT RNA fragment were incubated with or without each individual oligo, at a 1:1 molar ratio, in a 1.5 ml microcentrigue tube in 10 μl volume of 1× (final concentration) renature buffer (as above). Tubes were incubated to 95°C for 5 min and pulsed in the microcentrifuge to recover evaporated content. Reactions were then slow-cooled on the bench to 30°C. 2.5 μl of refolding buffer (as above) was subsequently added and the reactions were incubated at 37°C for 1h, Products were electrophoresed on ethidium bromide-containing 1% agarose gels in 1× TBM alongside the RbLR ladder (Thermo Fisher Scientific, #SM1833) at 70 V for 1h. Bands were visualized using UV transillumination. The monomeric and dimeric RNA bands were subsequently analysed and quantified using densitometry (ImageJ: https://imagej.nih.gov/ij/index.html).

#### Constructs and primers used

Agilent QuikChange Primer Design Program (https://www.agilent.com/store/primerDesignProgram.jsp) was used to create primer sequences for site directed mutagenesis (SDM). Primers were as follows:

S1Fw, 5′ GAGTGCTTCAAGTAGTGTGCACCCATCTGTTGTGTGACTCTGGT 3′;

S1Rv, 5′ ACCAGAGTCACACAACAGATGGGTGCACACTACTTGAAGCACTC 3′;

U5sFw, 5′ GCCTTGAGTGCTTCAAGTAGTGCGCACCCATCTGTTGTGTGACTCTGGTAAC 3′;

U5sRv, 5′ GTTACCAGAGTCACACAACAGATGGGTGCGCACTACTTGAAGCACTCAAGGC 3′; LU5AUGFw1 5′ CTAGCGGAGGCTAGAAGGAGAGAGACGGGCACAAGAGCGTCAGTAT 3′; LU5AUGRv1 5′ ATACTGACGCTCTTGRGCCCGTCTCTCTCCTTCTAGCCTCCGCTAG 3′;

LU5AUGFw2 5′ GCTAGAAGGAGAGAGACGGGCACAAGAAAAAAGGTATTAAGCGGGGGAGA

ATTAGATCGC 3′;

LU5AUGRv2 5′ GCGATCTAATTGTCCCCCGCTTAATACCTTTTTTCTTGTGCCCGTCTCTCTCCTTC

TAGC 3′;

A 2790 bp fragment from the WT pCCL-eGFP BglG and MS2 transfer vectors (38) containing the sequences between the CMV and hPGK promoters was amplified in a PCR reaction using the 5′-TACGGTAAACTGCCCACTTG-3′ and 5′-TAGGTCAGGGTGGTCACGAG-3′ primers in the AccuprimeTM *Pfx* Supermix (Thermo Fischer, #12344040). The 2790 bp PCR product was cloned into the pCR™Blunt II-TOPO^®^ vector (Invitrogen, #K280002) as per manufacturer′s instructions and was subsequently used to transform Stbl3 *Escherichia coli* competent cells (Invitrogen, #C737303). Mutagenesis was performed using the Site-Directed-Mutagenesis (SDM) XLII Agilent kit (#200521) using the primers presented above. Mutations were verified by Sanger sequencing. pCCL-eGFP BglG and MS2 transfer vectors and the resulting mutant pCR™Blunt II-TOPO® vectors were digested with NdeI and MfeI-HF and the mutated 1081 bp NdeI–MfeI fragment was ligated back into the digested 6729 bp NdeI–MfeI pCCL-eGFP BglG and MS2 backbones to produce the mutant transfer vectors presented in Figure 1C.

#### Lentiviral vector production by transient transfection

Production of third generation lentiviral vectors was performed as previously described (38). Briefly 5 × 10^6^ 293T cells maintained in Dulbecco's modified Eagle's medium (DMEM) (GIBCO, #10-569-044) supplemented with 10% fetal bovine serum (GIBCO, #10082147) (cDMEM) were seeded in 10 cm^2^ dishes in a final volume of 10 ml cDMEM. Fresh cDMEM was added 18 h later and cells were transfected 2–3 h later. For each transfection plate, 500 μl of 250 mM CaCl_2_ containing a total of 25 μg DNA in a 1:1:1:3 molar ratio of pMDLg/pRRE (Addgene, #12251): pRSV-Rev (Addgene, #12253):pMD2.G (Addgene, #12259): pCCL-eGFP transfer vector was added dropwise to 500 μl of 2× HEPES Buffered Solution pH 7 (Alpha Aesar™, #J62623AK) whilst bubbling air through it. Precipitates were incubated at room temperature for 30 min before being added dropwise across the plate. 5 ml of fresh cDMEM were added 16 h post transfection, cells were harvested at 40 h post transfection, aliquoted into cryovials, and stored at –80°C. Transfection efficiency was assessed by flow cytometry detecting eGFP expressing cells. The minimum transfection level for continuation of experiments was 95%.

#### Vector titration with flow cytometry

Viral vector transduction efficiency was titrated as described previously (38). Briefly, 3 × 10^5^ 293T cells were seeded per well of a six-well plate in a final volume of 2 ml. Frozen viral vector supernatants were thawed and used to transduce 293T cells 18 h post-seeding in 500 μl cDMEM containing 8 μg/ml polybrene. Lentiviral vectors were titrated 48 h post-transduction by flow cytometry assessment of eGFP positive cells. Titres were calculated using wells with transduction efficiencies ranging between 4% and 25%. The transduction efficiency of each lentiviral vector was calculated by multiplying the number of seeded cells by the percentage of eGFP positive cells and by the vector dilution factor and divided by the volume of the transduction culture. Transduction units were defined as the number of functional viral particles in the supernatant that were capable of transducing a cell and thus successfully driving the expression of the eGFP transgene.

#### RNA purification

Intracellular RNA from transfected cells was extracted using the Qiagen RNeasy kit (#74004) as described by the manufacturer. Extracellular RNA extraction from lentiviral particles was performed as previously described (38).

#### RT-qPCR assay

Relative packaging efficiency was measured using a previously published competitive RT-qPCR assay developed as per the MIQE guidelines (38). Briefly, contaminant plasmid DNA was removed using the Turbo DNA-free™ kit (Invitrogen, #AM1907) in a reaction containing 8 U of Turbo DNase™ Enzyme, 1× Turbo DNase™ Buffer and the total amount of RNA isolated, at 37°C for a total of 2 h DNase treatment incubation time. RNA was subsequently incubated with one fifth volume of the inactivation reagent at room temperature for 5 min and was recovered after centrifugation at 10 000 × *g* for 2 min. Reverse transcription was performed using the High-Capacity cDNA Reverse Transcription kit™ (Applied Biosystems, #4368814) as per the manufacturer’s instructions. cDNAs were then quantified by qPCR using a common set of primers Fw 5′-GAATTCTGCAGTCGACGGTA-3′, Rv 5′-TCCAGAGGTTGATTGCGA-3′ and probes targeting either the unique sequence of BglG—5′-FAM-TCGATCGGGATTGTTACTG-BHQ1-3′ or MS2—5′-HEX-CATGGGTGATCCTCATGCCGAT-BHQ2-3′; sequences contained in the backbone of all transfer vectors. The final reaction conditions were 750 nM BglG probe or 250 nM MS2 probe, 500 nM each primer, 1xTaqMan™ Fast Advanced Master Mix (Applied Biosystems, #4444556) and 3 μl cDNA in a 10 μl reaction volume. Standard curves were built using plasmids containing a single copy of the BglG and MS2 unique sequences. Samples with Ct values 30–35 were rejected to ensure measurements were restricted to the linear part of the standard curve and samples were considered negative when Ct values were higher than 35.

#### Northern blotting

RNA visualization was achieved as previously described (38) using a 744 nucleotide long DIG-labelled RNA probe that targets the end of the eGFP ORF and the start of the WPRE sequence. Extracellular RNA samples were purified as described above and analysed on ethidium bromide-stained 1% agarose gels in 0.5× UltraPure™ Tris–borate–EDTA (TBE) (Thermo Fisher Scientific, #15581044) and electrophoresed alongside the RiboRuler High Range RNA Ladder (RbHR ladder) (Thermo Fisher Scientific, #SM1821) at 60 V for at least 4 h. Marker bands were visualized using UV transillumination and their relative positions within the gel noted. 2 h capillary transfer in UltraPure™ 20× SSC (Thermo Fisher Scientific, #15557036) promoted RNA binding of samples from the 1% agarose gel to a Hybond N+ nylon membrane (GE Healthcare, #RPN203B). The membrane was subsequently baked at 80°C for 30 min and hybridized overnight at 68°C in UltraHyb (Invitrogen, #AM8670) containing 75 ng of the DIG-labelled probe. The DIG-labelled RNA-bound probe was targeted using anti-DIG-AP fragments antibody (Roche, #11093274910) in a 1:20 000 dilution and the DIG luminescent detection kit (Roche, #11363514910) following the manufacturer′s protocol. RNA was visualized with the addition of CDP-Star and detected with photographic film. Monomeric (3866 nt) and dimeric RNA (7732 nt) bands were aligned against the positions of the RbHR ladder. The lower band on the presented northern blots was defined as the monomeric vector RNA band as it aligned with the second top band of the RbHR ladder (4000 nt). The top band on the presented northern blots was defined as the dimeric vector RNA band as it was visualized to be above the top band of the RbHR ladder (6000 nt). The monomeric and dimeric RNA bands were subsequently analysed and quantified using ImageJ (https://imagej.nih.gov/ij/).

#### ELLA p24 assay

p24 quantification was achieved using the ELLA simplex immunoassay (ProteinSimple) according to the manufacturer′s instructions. Aliquots of frozen harvested supernatant were thawed and diluted 500x in 0.5% Triton X Lysis buffer (ProteinSimple). 50 μl of the diluted samples were loaded on the ELLA cartridge (ProteinSimple) with 1 ml of the Wash Buffer (ProteinSimple) loaded in each designated well. Each run was 90 min duration and the integral software automatically calculates the p24 amount per well (pg/ml) using an inbuilt standard curve. Information about the HIV-1 Gag p24 ELLA simplex immunoassay and reagents used are available online at https://www.proteinsimple.com/documents/HIV-1_Gagp24_Specification_Sheet_D10-1145-001_Rev_A.pdf

#### Reagents

Phusion DNA polymerase (NEB, # M0530S) NEB Monarch extraction kit (#T1020S), MEGAScript T7 transcription kit (Thermo Fisher, #AM1334), the RiboRuler Low Range RNA ladder (Thermo Fisher Scientific, #SM1833), Reverse transcription mix (Invitrogen, #18080044), Thermo Sequenase Cycle Sequencing Kit (Applied Biosystems, #785001KT), AccuprimeTM *Pfx* Supermix (Thermo Fischer, #12344040), (Invitrogen, #K280002), Site-Directed-Mutagenesis (SDM) XLII Agilent kit (#200521), DMEM (GIBCO, #10-569-044), fetal bovine serum (GIBCO, #10082147), 2× HEPES buffered solution pH 7 (Alpha Aesar™, #J62623AK), Qiagen RNeasy kit (Qiagen, #74004), Turbo DNA-free™ kit (Invitrogen, #AM1907), High-Capacity cDNA Reverse Transcription kit™ (Applied Biosystems, #4368814), 1xTaqMan™ Fast Advanced Master Mix (Applied Biosystems, #4444556), UltraPure™ TBE (Thermo Fisher Scientific, #15581044), RiboRuler High Range RNA Ladder (Thermo Fisher Scientific, #SM1821), UltraPure™ 20× SSC (Thermo Fisher Scientific, #15557036), Hybond N+ nylon membrane (GE Healthcare, #RPN203B), UltraHyb (Invitrogen, #AM8670), anti-DIG-AP fragments antibody (Roche, #11093274910), DIG luminescent detection kit (Roche, #11363514910)

#### Biological resources

Stbl3 *E. coli* competent cells (Invitrogen, #C737303), pCCL-eGFP (Addgene, #10880), pMDLg/pRRE (Addgene, #12251): pRSV-Rev (Addgene, #12253): pMD2.G (Addgene, #12259)

#### Computational resources

ImageJ (https://imagej.nih.gov/ij/), RNAstructure software (https://rna.urmc.rochester.edu/), XRNA (http://rna.ucsc.edu/rnacenter/xrna/), SHAPEfinder software (https://github.com/drsuuzzz/ShapeFinder), Agilent QuikChange Primer Design Program (https://www.agilent.com/store/primerDesignProgram.jsp), Los Alamos HIV Sequence Database (http://www.hiv.lanl.gov/), BLAST (https://blast.ncbi.nlm.nih.gov/Blast.cgi)

#### Statistical analyses

Independent two sample *t* tests with a two-tailed distribution were used for all statistical analyses.

## RESULTS

### A U5–AUG stabilizing transfer vector mutant increases dimerization with neutral effects on packaging and negative effects on infectivity

**Figure 2.**
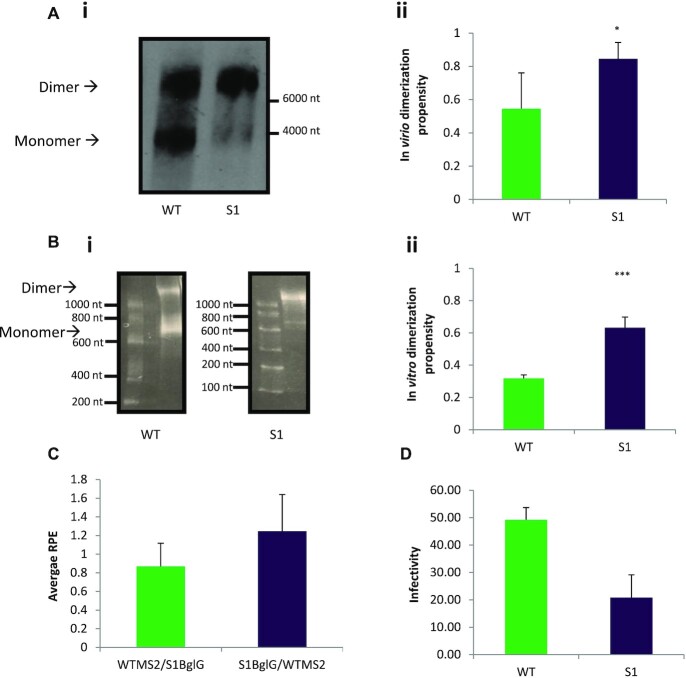
Biochemical characterization of WT and S1 transfer vector mutants. (**A**, i) Representative northern blot of WT and S1 vector RNAs. 293T cells were transfected with either WT or S1 transfer vector alongside the packaging plasmids, and RNA was extracted and purified from equal volumes of harvested viral supernatant. RNA was then electrophoresed on a TBE gel, size markers were visualized with Ethidium Bromide and their relative positions within the gel noted, blotted, and transfer vector RNA was visualized with a DIG-labeled probe. The positions of the 6000 and 4000 nt size markers are shown. The expected size of the monomer is 3866 nt and the dimer is 7732 nt. Blot is representative of three independent experiments. (ii) average dimerization from the three independent northern blot replicates, expressed as the amount of dimer as a percentage of the total RNA, *P*< 0.05, error bars show standard deviation (**B**, i) TBM gel from *in vitro* dimerization of 690 nt long WT and S1 RNA fragments containing sequences spanning R to *gag*. RNA size markers are shown. Image is representative of six independent replicates. (ii) average dimerization of the six independent replicates, expressed as the amount of dimer as a percentage of the total RNA (*P*< 0.01, error bars show standard deviation). (**C**) Average WT and S1 transfer vector RPE from eleven independent co-transfection repeats. RPE was measured by dividing the extracellular to intracellular RNA levels of S1 to the extracellular to intracellular RNA levels of WT in a co-transfection environment. Error bars show standard error. (**D**) Measurement of average infectivity (TU/24 pg) of the WT and S1 transfer vectors. Infectious titers (TU/ml) were calculated by flow cytometry of eGFP-expressing infected cells and physical titers were calculated by performing ELLA p24 on purified viral supernatants.

The role of the U5–AUG duplex in promoting a structure that favours dimerization *in vitro* has been reported by multiple groups (9, 10, 39). A previously designed mutant, S1 (9), characterized by increased base pairing in its U5–AUG duplex and by a 60% increased dimerization propensity *in vitro* (9), provided an appropriate candidate to study the relationship between dimerization, packaging and infectivity. To investigate the dimerization properties of the S1 mutant in an *in virio* context, the appropriate three point mutations were introduced into the backbone of the third generation lentiviral transfer vector plasmid (Figure 1C, ii). Wild-type and S1 transfer vectors were transfected independently into 293T cells with plasmids encoding the packaging constructs. Two days post transfection, vector particles were harvested and purified from supernatant and their RNA content analysed by northern blot to assess their propensity to dimerize *in virio* (Figure 2A, i). In agreement with previous studies (40, 41), this northern blot assay detected both monomeric and dimeric RNA species. HIV-1 dimerization is thought to involve an initial kissing-loop ‘loose’ dimer, progressing to a ‘tight’ dimer (15). The loose dimer is stabilized by magnesium and will separate back into monomers on a TBE gel. The ‘tight’ or ‘extended’ dimer is thought to be more stable in the absence of magnesium. As a TBE gel, our northern blot reports both on the ability of the dimer to form initially, and to stabilize. We thus use the term dimerization propensity to describe our results, as this takes into account both aspects of dimerization. Densitometric analysis of northern blot dimeric and monomeric RNA levels from three independent experiments showed that S1 vector RNA was characterized by increased *in virio* dimerization propensity compared to WT (*n* = 3 for both WT and S1, *P* = 0.04 by an independent two sample *t* test with a two-tailed distribution, Figure 2A, ii). *In vitro* assays replicated this result, showing twice as much dimerization in the S1 mutant (*n* = 4 for both WT and S1, *P* = 0.0009 by an independent two sample *t* test with a two-tailed distribution) using 690 nt long *in vitro* synthesized RNA fragments of the WT and S1 transfer vectors (Figures 2B, i and 2B, ii). These included the *cis*-acting elements of the HIV-1 5′ leader and the partial *gag* region: sequences established to be important for the maintenance of viral titers. This similarity in results confirms that these RNAs dimerize similarly under *in virio* and *in vitro* conditions. To see whether the increase in dimerization of the S1 mutant led to an increase in vector RNA encapsidation, a recently described novel competitive packaging assay was used (38), where 293T cells were co-transfected with equal amounts of WT and S1 transfer vectors alongside the packaging plasmids. Both intracellular and virion RNAs were extracted and purified to measure their relative packaging efficiency. This assay was chosen over others as its particular design allows for co-transfection of the WT and mutant transfer vectors, which intrinsically normalizes for any differences in transfection efficiency, that could be affecting LTR and internal promoter-driven transgene expression discrepancies. Additionally it eliminates skewing of results from potential splicing occurring between SD1, located in the 5′ leader, towards the 3′ end of the U5 region, and the SA7, present in the RRE*/env* sequences. Relative packaging efficiency (RPE) was defined as the ratio of extracellular to intracellular WT gRNA divided by the ratio of extracellular to intracellular S1 gRNA derived from the same co-transfection plate. S1 RPE was measured to be 1.2, which was not statistically significantly different from WT (*n* = 11, *P* = 0.55 by an independent two sample *t* test with a two-tailed distribution, Figure 2C) indicating that despite an increased dimerization capability, this transfer vector mutant has a similar packaging efficiency to WT. This result suggests that dimerization enhancement does not automatically lead to an increase in packaging. Finally, physical and infectious titers of vector supernatants were measured to study the effect of S1 mutants on infectivity. Independent transfections were performed as described above for northern blots, followed by serial dilutions of harvested lentiviral supernatant which were either used to transduce HEK293T cells or assayed for p24 Capsid content. The ability of the rationally designed transfer vector to successfully transduce cells and express the eGFP transgene was investigated with flow cytometry and measured in transduction units (TU/ml) as described in materials and methods. ELLA p24 was performed to measure Capsid-p24 protein concentration and thus Gag protein expression, which provides a measure of physical vector titer. Infectivity was defined as the average ratio of TU/ml to p24 pg/ml (TU/p24 pg) from 9 independent WT and S1 viral supernatants. Unexpectedly, as shown in Figure 2D, despite the increase of dimerization and neutral effects upon packaging the S1 vector mutant demonstrated a 50% reduction in infectivity (*n* = 9 both for WT and S1, *P* = 0.05 by an independent two sample *t* test with a two-tailed distribution), showing that a mutation that improves dimerization does not automatically improve packaging or infectivity.

### In-gel SHAPE reveals novel interactions that are dependent on the 3′ *gag* sequences

Overall, our data suggest that the mutations introduced into the U5 region affect not only the dimerization properties of HIV-1 derived lentiviral vector RNA, but also negatively influence viral infectivity by effects on genome packaging and possibly other undefined processes in the lifecycle. The S1 transfer vector was designed to increase lentiviral vector efficiency based on existing structural information linking stabilization of U5–AUG and increased dimerization.

The roles of *cis*-acting elements included in the backbone of the third generation lentiviral transfer vector system have been extensively studied (42), however the molecular mechanisms that underlie the function of the partial *gag* sequence remain unknown. Efforts to shorten or remove it completely have led to decreased packaging efficiency (19–23). Thus pragmatically a partial *gag* fragment of 361 bp (24) has been maintained in the transfer vector design (Figure 1B, ii). We therefore decided to investigate the impact of the S1 mutations in the longer RNA that is used empirically for maximal RNA encapsidation in lentiviral vectors.

**Figure 3.**
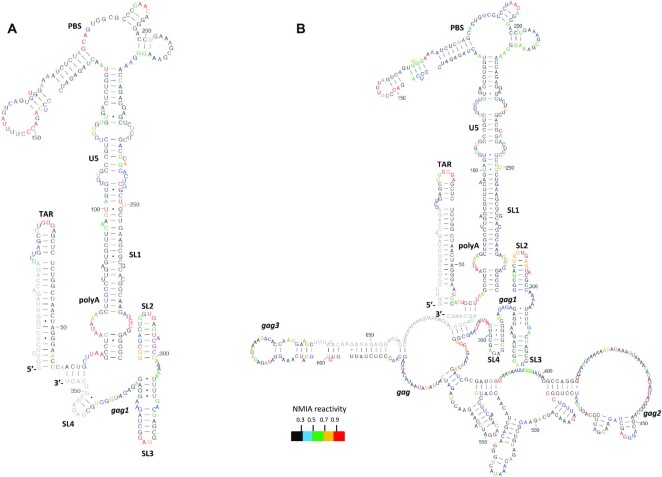
Proposed structures of monomeric WT HIV-1 leader RNA with or without the presence of the partial *gag* sequences. (**A**) Model derived using in-gel SHAPE data from monomeric WT HIV-1 RNA lacking the extended *gag* sequences. (**B**) Model derived using in-gel SHAPE data from monomeric WT HIV-1 RNA containing the extended *gag* sequences. All RNAs were renatured, electrophoresed, in-gel probed and modelled as described in Materials and Methods. Single nucleotide level average NMIA reactivities are shown by colour according to the key and are mapped onto each proposed structure. NMIA data are an average of three independent experiments for WT RNA lacking *gag* sequences and of four independent experiments for the *gag* containing WT RNA. *gag1* corresponds to the first 21 highly conserved nucleotides of the Gag ORF. *gag2* is defined as the region of *gag* found to interact with the U5 sequences of the HIV-1 leader. *gag3* is defined as the *gag* region found to interact with the polyA sequences of the HIV-1 leader. *gag* corresponds to nucleotides 335–690 of the HIV-1 HXB2 sequence.

**Figure 4.**
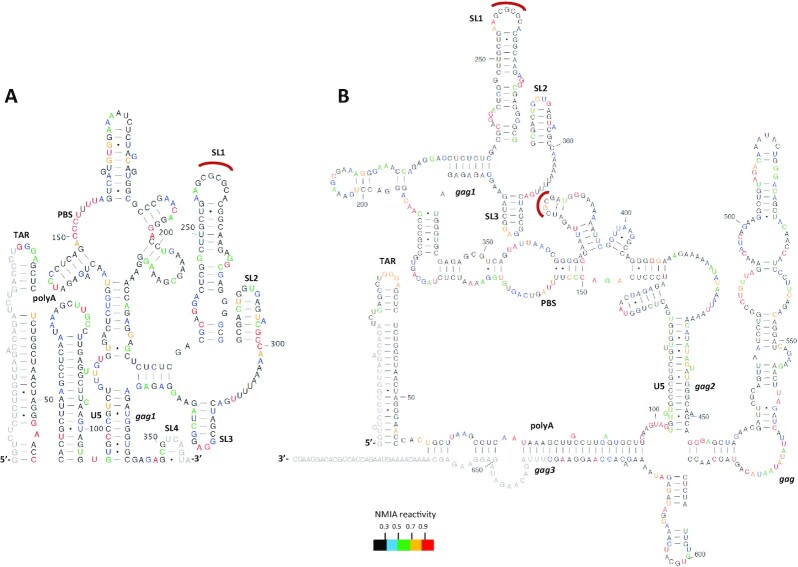
Proposed structures of dimeric WT HIV-1 leader RNA with or without the presence of the partial *gag* sequences. (**A**) Model derived using in-gel SHAPE data from dimeric WT HIV-1 RNA lacking the extended *gag* sequences. Intermolecular sites are depicted with a red curve. (**B**) Model derived using in-gel SHAPE data from dimeric WT HIV-1 RNA containing the extended *gag* sequences. Intermolecular sites are depicted with a red curve. All RNAs were renatured, electrophoresed, in-gel probed and modelled as described in Materials and Methods. Single nucleotide level average NMIA reactivities are shown by colour according to the key and are mapped onto each proposed structure. NMIA data are an average of three independent experiments for WT RNA lacking *gag* sequences and of four independent experiments for the *gag* containing WT RNA. *gag1* corresponds to the first 21 highly conserved nucleotides of the Gag ORF. *gag2* is defined as the region of *gag* found to interact with the U5 sequences of the HIV-1 leader. *gag3* is defined as the *gag* region found to interact with the polyA sequences of the HIV-1 leader. *gag* corresponds to nucleotides 335–690 of the HIV-1 HXB2 sequence.

In gel-SHAPE is a technique that enables structural modelling of individual conformers within a mixed structural population, under native conditions and without using mutagenesis (10). Here, it was employed to study the effect of the extended *gag* sequences on *psi* by comparing the monomeric and dimeric RNA structures adopted by the transfer vector RNA with those formed by a shorter RNA that lacks the majority of this *gag* sequence. Such shorter RNAs have previously been studied by multiple structural techniques (9,10,13,39). 416 nt long RNA fragments containing the cis-acting elements of the HIV-1 leader and 79 nt of the *gag* region, and 690 nt long RNA fragments including both the HIV-1 leader and the adjacent *gag* sequences were prepared by *in vitro* transcription. The respective RNAs were renatured, refolded and then fractionated by native gel electrophoresis to separate the monomeric and dimeric RNA. Individual RNA species were isolated and probed with NMIA within the gel, recovered by electroelution and reverse transcribed to map NMIA binding sites. The most thermodynamically favorable structures and their single-nucleotide level NMIA reactivities are presented in Figures 3 and 4, alongside Supplementary Table S1. As shown in Figure 3A, under the refolding conditions used here, the shorter RNA monomer is stabilized into a structure resembling the LDI (long distance interaction) model (9) around the poly(A)- DIS region, with some reactivity at the base of the TAR stem that suggests some flexibility in that region and also the possible presence of a wider structural ensemble. The monomer of the longer RNA folded very similarly across the 5′ nucleotides common to both RNA lengths (Figure 3B). The SHAPE reactivity differences in nucleotides contained in both short and longer RNA monomers were minimal (5% of nts had a *P* value below 0.05 by *t* test (data not shown), which is the level that would be expected by random chance) and is not suggestive of a large-scale structural rearrangement. The *gag* sequences absent from the shorter RNA were found within self-contained structures in the longer RNA and no long-range interactions were predicted. Thus the structural features underlying the enhanced packaging ability of the longer RNA do not appear to be due to the structural influence of the 3′ sequences upon the major packaging signal region in the monomeric species.

Overall the dimeric structure of the short monomer presented in Figure 4A was similar to previously proposed BMH (branched multiple hairpin) (9)-like structural models, containing the U5–AUG helix (9, 10). However in the dimeric RNA formed from the two longer monomers that included extended *gag* sequences an unexpected structural rearrangement occurred. Whilst the presence of the U5–AUG motif in the dimeric RNA of the WT HIV-1 leader has been supported by several different groups (9, 10, 13), when probed by in-gel SHAPE, strikingly, in the presence of the extended *gag* sequences there was no U5–AUG formation in the dimer. Instead a novel direct interaction was identified between the U5 region (nts 103–122) and a sequence of *gag* nucleotides that are not present in the shorter RNA (nucleotides 433–452, hereafter referred to as *gag2*; Figure 4B). In addition, unlike the formation of the polyA stem loop noted in the shorter dimeric RNA, sequences of the polyA (61–95nt) were observed to pair with nucleotides of the *gag* region (621–660 nt, hereafter referred to as *gag*3; Figure 4B) that are also unique to the longer RNA. *gag1* (Figure 4B) corresponds to the first 21 nucleotides of the Gag open reading frame (ORF) known to contain a highly conserved sequence that has been reported to be incorporated into the genomic RNA dimer structure (39), *gag* corresponds to nucleotides 335–690 of the HIV-1 HXB2 sequence that were added for further investigation in this study. Addition of *gag* sequences did not affect the formation of the PBS and SL1–SL2 structural motifs, which formed as described above. However, an effect on the reactivity values of SL3, denoted as the ψ element, was evident. In this predicted dimeric structure, SL3 was formed by nucleotides 312–325. The majority of the nucleotides (10/14) forming SL3 showed a reduced reactivity value. In contrast three of them, one from the loop region of SL3 and two from the lower part of the stem, were characterized by an increase in their measured NMIA reactivity. One base showed no change in reactivity.

Whilst the monomeric structure contained the extended SL3 helix the dimeric structure did not. This observation suggests that *gag* sequences stabilize the ψ element in the dimeric RNA and change the structures of the nucleotides immediately 5′ and 3′ of it. The longer *gag*-containing dimeric RNA structure was not characterized by the formation of SL4 despite the lack of formation of the U5–AUG duplex. Overall, addition of the *gag* sequences present in an optimal length transfer vector RNA had no effect on the monomeric structure of the 5′ region but results in two direct interactions between *gag* and the HIV-1 leader, including the polyA and U5 regions, that form in the dimeric RNA *in vitro*.

### The novel long-range interactions identified by in-gel SHAPE are structurally conserved amongst HIV-1 isolates

Our in-gel SHAPE structural prediction suggested that the most stable dimeric structure of the longer RNA contains a novel interaction between U5 nucleotides and 3′ *gag* sequences, rather than the formation of the highly conserved and well documented U5–AUG helix. If these interactions are functionally important to HIV-1, then they would be expected to be structurally conserved amongst various naturally occurring HIV-1 isolates. To investigate the likelihood of the U5–*gag* interaction being of biological importance, sequence and structure conservation analysis was performed for nucleotides 103–122 (U5) and (433–452) (*gag*) by comparison of all HIV-1 Group M strains from the Los Alamos database sequence compendium database 2018 (http://www.hiv.lanl.gov/). In order to put our findings into context we performed the same analysis for the U5–AUG interaction (U5 nucleotides 105–115 and *gag* nucleotides 334–344). All three sequences involved are highly conserved, but do still contain some sequence variants. Our findings presented in Table 1 suggest that where these variants occur, the predicted structures they form are preserved, with a very similar degree of predicted structural conservation observed for both interactions, an average of 97.4% of base-pairs forming for U5–AUG and 97.04% for U5–*gag*, which is a longer interaction. All strains were able to form a substantial part of each predicted structure, with 55 out of 57 strains able to form at least 10 of 11 base-pairs of U5–AUG and 54 out of 57 strains able to form at least 16 of 18 base-pairs of U5–*gag* structural motifs. One out of the 57 was an outlier, capable of forming less than half of base-pairs in both the U5–AUG and U5–*gag* predicted structures (02_AG.PK.15.PK032). This virus was derived from an incompletely characterized patient source with a low viral load from an analysis of recombinant CRF02/A1 viral spread in Pakistan from a point source introduction and would need full sequence analysis to investigate if there are other sequence characteristics that might compensate structurally (43). The equivalent levels of phylogenetic conservation capable of generating predicted specific structures between the two interactions suggests the importance of both sequences in the HIV-1 lifecycle. Additionally, to further interrogate the biological importance of the U5–*gag* interaction, base pair co-variation analysis was performed for both U5–AUG and U5–*gag* putative interactions by comparison of all HIV-1 Group M strains. We have taken a G–U wobble pair as an equivalent to a G–C pair in terms of geometrical ability to form a duplex notwithstanding the difference in hydrogen bond number. As shown in Supplementary Table S2, 19 base pair semi co-variations (A–U or G–C to G–U or vice-versa) were noted for the U5–*gag* interaction amongst 15 different HIV-1 strains out of 178 group M strains analysed, compared to 16 base pair semi co-variations found within 12 different strains of the 178 analysed for the U5–AUG interaction, further supporting the importance of the novel U5–*gag* interaction, particularly since the *gag* sequence of the U5–gag interaction is constrained by coding and the AUG side of the U5–AUG helix is only partly coding. The polyA–*gag* interaction was interrogated by comparing the stability of the predicted polyA and polyA–*gag* structural motifs amongst the frequently occurring polyA sequence variations of all M strains. As shown in Supplementary Figure S1 polyA–*gag* was noted to be conserved in terms of always forming a predicted stabilizing structure albeit not always forming the same specific structure. Its importance is likely to be in stabilizing the overall RNA structure of the U5–*gag* containing dimer, rather than performing a separate, specific function of its own. These findings of structural conservation of the U5–*gag* interaction and the stabilizing nature of the polyA–*gag* interaction are particularly significant given that parts of the HIV-1 life cycle, including gRNA packaging, depend on structural motifs in the RNA rather than sequence recognition (44).

**Table 1.** Phylogenetic analysis of the U5–AUG and U5–*gag* interactions. Comparison of all 178 isolates that belong to group M. Sequence conservation indicates the % of sequences with a variation from the reference HXB2 strain at each nucleotide position. Structural conservation indicates the % of isolates in which the base-pairing nature at each nucleotide position is maintained. ‘–’ denotes unpaired nucleotides. [A] Study of sequence and structure conservation of the previously reported U5–AUG duplex. [B] Study of sequence and structure conservation of the newly observed U5–*gag* interaction

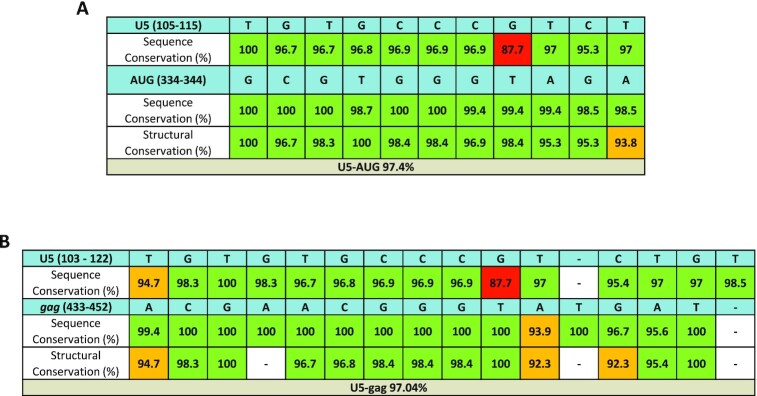

### The S1 mutant monomeric and dimeric structures contain the U5–AUG motif, when extended *gag* sequences are present

**Figure 5.**
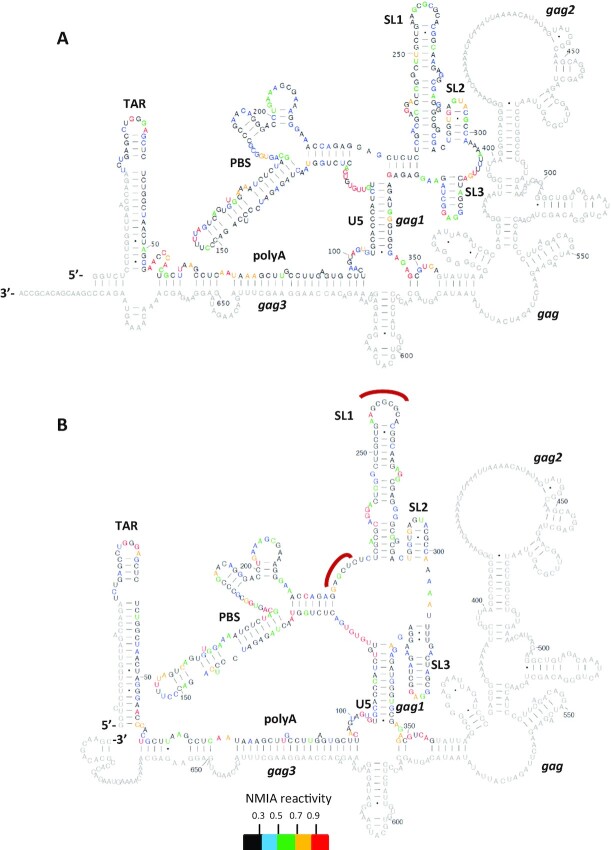
Structural models of monomeric and dimeric S1 HIV-1 leader RNA. (**A**) Model derived using in-gel SHAPE data from monomeric S1 HIV-1 RNA containing the extended *gag* sequences. (**B**) Model derived using in-gel SHAPE data from dimeric S1 HIV-1 RNA containing the extended *gag* sequences. Experiments were conducted and models depicted as described in Figures 3 and 4. NMIA data are an average of four independent experiments. Intermolecular sites are depicted with a red curve.

Next, we sought to determine the effect of the introduced S1 mutations on RNA structure to further probe the relationship between RNA structure and dimerization of the vector RNA. We performed in-gel SHAPE as above on a 690 nt long *in vitro* dimerized transcript that contained the S1 mutations and the extended *gag* sequences. Interestingly as shown in Figure 5A, the long S1 monomeric RNA exhibited several structural similarities with the long WT dimeric RNAs. The NMIA reactivity data mapped accurately on to previously described TAR structures. However, unlike the longer WT monomeric RNA that was characterized by the formation of polyA, the long S1 monomeric RNA was characterized by a polyA–*gag* interaction, a feature only previously observed in the longer WT dimeric RNA. Downstream of the polyA–*gag* interaction, in the S1 long RNA monomer we observed the formation of the U5–AUG structural motif, previously described in the shorter WT dimeric RNA that did not contain the *gag* sequences. Strikingly, the formation of SL1 was also observed in the long S1 monomeric RNA, a structural element commonly featured in dimeric RNAs. The remaining data mapped accurately onto the PBS, SL2 and SL3 structures of the long S1 monomeric RNA. The formation of the U5–AUG and SL1 structural motifs observed in this monomeric S1 RNA had so far been described as characteristic features of the secondary structures of dimeric RNAs. The predicted structure suggests that the introduced S1 mutations indeed strengthened the U5–AUG interaction and promoted characteristics of the dimerization-competent structure, including the formation of SL1 and exposure of the DIS, in the monomer.

Long S1 dimeric RNA NMIA reactivity data mapped accurately on the structure of TAR and on the polyA–*gag* interaction as predicted for the long WT dimeric RNA secondary structure. However, unlike the U5–*gag* interaction observed in the long WT dimeric RNA, S1 long dimeric RNA was characterized by the formation of a U5–AUG structural motif. Notably, the S1 mutant was designed to have a strengthened U5–AUG interaction, which as presented in Figures 5A and B, was formed both in the monomeric and dimeric long S1 RNAs. PBS, SL1-SL3 fitted the SHAPE data from the long WT RNA accurately, with the exception of reactivity differences at the base of SL3. S1 and WT long dimeric RNAs were shown to have very similar secondary structures with the exception of the U5–*gag* interaction of WT being substituted with the formation of the U5–AUG structural motif in S1. Overall this data highlights the previously reported role of U5–AUG in promoting a dimerization competent structure.

### Interrogation of the RNA structure of further U5–AUG mutants with in-gel SHAPE suggests an expanded structural equilibrium

**Figure 6.**
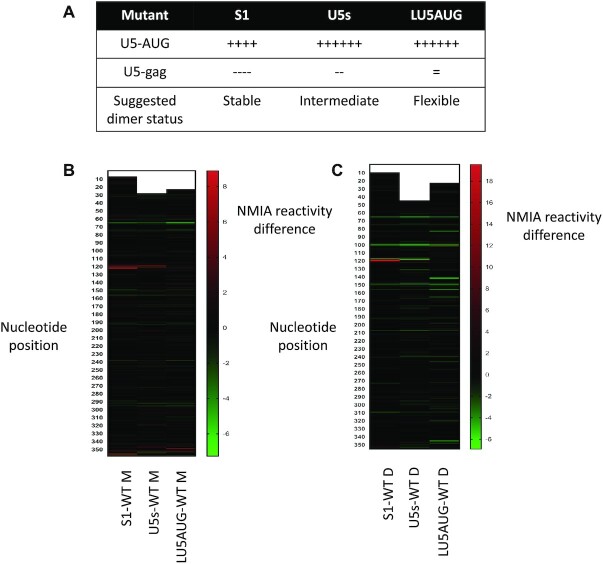
Effect of U5–AUG mutations on local structure and NMIA reactivity. (**A**) Structural and dimerization characterization of U5–AUG mutants. (**B**) Heat map presenting NMIA reactivity difference between mutant and WT monomeric RNA. (**C**) Heat map presenting NMIA reactivity difference between mutant and WT dimeric RNA. All NMIA reactivities interrogated here are derived from 690 nt *gag* containing RNAs.

To further evaluate the presence of this newly observed U5–*gag* interaction we designed more mutants to alter the relative stabilities of the U5–AUG helix and the U5–*gag* interaction. Nucleotide changes to the U5 side of the helix would be predicted to affect both structures, but with potentially different impacts upon each, and changes to the *gag* side of the helix could be designed to have a more targeted impact on a single structure. As shown in Figure 1C and 4B, the wild-type U5–AUG helix is stabilized by 23 Watson–Crick or G–U wobble hydrogen bonds and the novel U5–*gag* interaction by 39 Watson–Crick or G–U wobble hydrogen bonds across the extended region, separated by several bulged or unpaired nucleotides. The S1 transfer vector mutant contained 3 point mutations to the U5 side that stabilize the U5–AUG helix by four hydrogen bonds (Figure 1C, ii) and destabilize the U5–*gag* interaction by four hydrogen bonds. The U5s mutant stabilizes the U5–AUG helix by six hydrogen bonds (Figure 1C, iii) and destabilizes the U5–*gag* interaction slightly, by two hydrogen bonds. The LU5–AUG mutant (39) also stabilizes the U5–AUG helix by 6 hydrogen bonds (Figure 1C, iv), but has no effect on the stability of the U5–*gag* interaction. All three mutants are thus predicted to skew the structural equilibrium towards the BMH structure, but with differing contributions towards the stabilization/destabilization of each of the two interactions as shown in Figure 6A.

The U5s and LU5AUG RNAs were *in vitro* transcribed and probed via in-gel SHAPE, as above. Focusing on the dimeric RNAs, in-gel SHAPE data now suggested that all three mutants were forming identical BMH-like structures, supported by the formation of the U5–AUG helix. Although the mutants contained only three or four point mutational differences from wild-type, SHAPE reactivities differed at several sites along the RNA, with a major reactivity difference concentrated at the GU rich region at nts 116–121 (Figure 6B and C). This is largely base-paired and unreactive in the dimeric model containing the U5–*gag* interaction but is unpaired and highly reactive in the BMH model. Therefore, LU5AUG, which has an intact U5–*gag* structural motif, lacks the observed increased reactivity at the GU-bulge noted for the S1 and U5s mutants that contain U5–*gag* destabilizing mutations.

**Figure 7.**
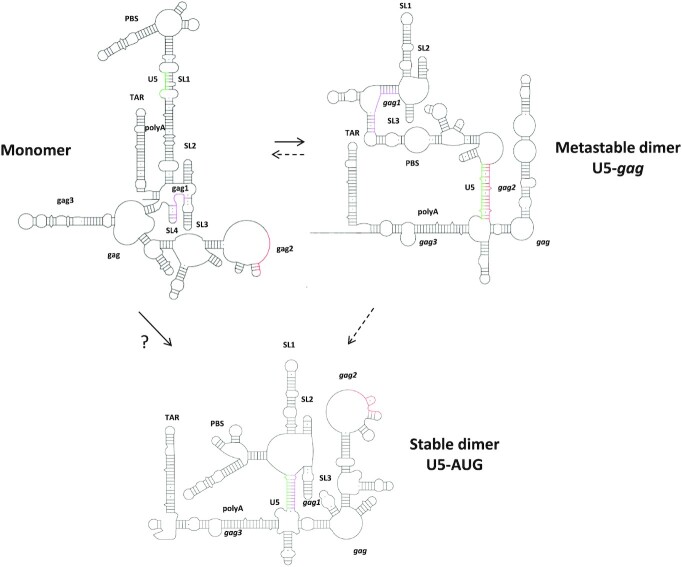
Proposed mechanism of an expanded structural equilibrium. Transition of the monomeric vector RNA to a stable dimeric infectious state is mediated by a structural equilibrium of at least two different structural states; one that is characterized by the formation of the newly observed U5–*gag* interaction, associated with a metastable dimeric conformation, and another one adopting the previously reported U5–AUG duplex known to form a stable dimeric conformation. The novel structural equilibrium exists between the monomeric conformation and the U5–*gag* and U5–AUG dimeric conformations. Whether the U5–*gag* structure reverts to the monomeric structure before adopting the U5–AUG dimeric structure or whether it is a true structural intermediate in the pathway between monomeric RNA and mature dimeric RNA is unknown. Dotted lines represent these possible pathways. The ‘‘?’’ represents the possibility of more than one structural step in the pathway.

Taking all these structural data together, our results suggest that the dimeric vector RNA exists in an equilibrium of at least two different structural states; one that is characterized by the formation of the newly observed U5–*gag* interaction, associated with a metastable dimeric conformation, and another one adopting the previously reported U5–AUG duplex known to form a stable dimeric conformation. Thus, here we suggest a novel structural equilibrium between the monomeric conformation and the U5–*gag* and U5–AUG dimeric conformations associated with different dimerization propensities, which are both essential for the transition of the monomeric vector RNA to a stable dimeric infectious state and that can explain the requirement for the partial *gag* sequences for efficient viral packaging (Figure 7). Additionally, this model, including a structural equilibrium of multiple conformations suggests that RNA flexibility plays an important role in the successful production of lentiviral vector particles.

### The structural equilibrium between U5–AUG and U5–*gag* influences the dimerization and encapsidation levels of the transfer vector RNA

The proposed structural equilibrium suggests that destabilization of the U5–*gag* interaction and/or stabilization of the U5–AUG structural motif could lead to increased dimerization. Based on this premise we hypothesized that the dimerization propensity of mutants should be as follows: S1 > U5s > LU5AUG > WT. To assess our hypothesis we performed biochemical assays to investigate the relationship between dimerization, packaging, infectivity and structural flexibility of the transfer vector RNAs.

**Figure 8.**
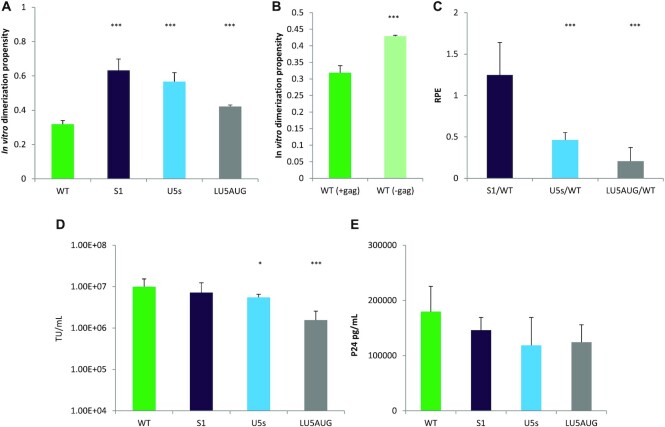
Functional assessment of U5–AUG mutants. (**A**) Densitometric analysis of *in vitro* dimerization efficiency of 690 nt long WT and U5–AUG mutants. Dimerization efficiency was measured as the average relative ratio of dimeric RNA to total levels of RNA from seven WT, six S1, seven U5s and three LU5AUG independent repeats. Error bars show standard deviation. (**B**) Dimerization efficiency comparison between *gag* containing and *gag* lacking WT RNA. Graph depicts average values from seven *gag* containing and three *gag* lacking RNA replicates. Error bars show standard deviation. (**C**) Measurement of WT and mutant transfer vector average RPE. Average RPE was measured from eleven WT-S1, eighteen WT-U5s and three WT-LU5AUG independent co-transfection repeats. Error bars show standard error. (**D**) 293T cells were transduced with varying dilutions of WT and mutant transfer vectors as described above in triplicates. Functional titers were calculated by flow cytometry of eGFP-expressing cells, using the dilution in which samples contained 4–25% eGFP+ cells. Error bars show standard deviation. (**E**) ELLA p24 was performed on purified WT and mutant viral vector supernatants. Physical titers were calculated based on measured p24 pg/mL from four independent replicates for each transfer vector. Error bars show standard deviation.

As shown in Figure 2A and B, S1 RNA was characterized by an increased dimerization propensity compared to WT both *in vitro* and *in virio*. Our previous studies had shown that there was around 17% more dimer in the U5s virions than WT virions (38). *In vitro* dimerization studies had to be performed due to the inability to detect LU5AUG vector RNA with northern blots, presumably due to the lower levels of total RNA packaged by this transfer vector (northern blot data not shown), and as suggested by our proposed equilibrium mechanism. *In vitro* dimerization studies (Figure 8A) were performed as above and showed that there was 2 times more dimer for S1 than WT (*n* = 7 for WT and *n* = 6 for S1, *P* = 0.00003), 1.8 times more dimer for U5s than WT (*n* = 7 for both WT and U5s, *P* = 0.000003) and 1.3 times more dimer for LU5AUG than WT (*n* = 7 for WT and *n* = 3 for LU5AUG, *P* = 0.000004 all by independent two sample *t* tests with a two-tailed distribution as described in Methods), confirming our hypothesis that U5–*gag* destabilization and U5–AUG strengthening leads to increased dimerization propensity and supporting the proposed structural equilibrium. Additionally, based on this suggested equilibrium we hypothesized that removal of the *gag* sequences from the WT RNA template should lead to an increase of *in vitro* dimerization propensity as consequentially WT RNA would only be able to adopt the more stable U5–AUG dimeric conformation. As shown in Figure 8B, removal of *gag* sequences indeed increased the *in vitro* dimerization propensity of WT by 1.3 times (*n* = 7 for WT(*+gag*) and *n* = 3 for WT(–*gag*), *P* = 0.000005 by an independent two sample *t* test with a two-tailed distribution). Antisense DNA oligos targeting the putative polyA–*gag* (o1) and the U5–*gag* (o2) interactions were designed as a final approach to investigate the presence of these two suggested novel interactions *in vitro*. Specific antisense DNA oligonucleotide targeting of RNA sequences has been extensively used as a method of studying structural features, RNA–RNA and RNA–protein interactions within the HIV-1 genome *in vitro* (37, 45–47). We hypothesized that if the polyA–*gag* and U5–*gag* interactions indeed occur, addition of these antisense oligos would hinder the formation of these structural motifs and lead to decreased dimerization *in vitro*. Oligonucleotides were designed to target the polyA–*gag* interaction by binding to *gag*2 and the U5–*gag* interaction by binding to *gag*3 (shown in Figure 4B), thus testing the effect of these structural aspects of the extended *gag* sequences upon dimerization. Oligonucleotides chosen were not predicted to bind elsewhere along the RNA and were incubated with it at a 1:1 molar ratio to minimize off-target interactions. Results are shown in Supplementary Figure S2. Incubation of the WT long RNA fragment with the o1 oligo led to a 20% reduction of dimerization propensity (*n* = 3 for WT control, *n* = 3 for WT + o1, *P* = 0.01), while incubation of the WT long RNA fragment with the o2 oligo led to a 25% reduction of dimerization propensity (*n* = 3 for WT control, *n* = 3 for WT + o2, *P* = 0.02). A scrambled oligo that was predicted not to interact with the RNA was added at the same molar ratio, did not affect dimerization (*n* = 4 for WT control, *n* = 4 for WT + scramble, *P* = 0.74, data not shown). These data further support the formation of the suggested polyA–*gag* and U5–*gag* structural interactions *in vitro*.

To further investigate the properties of the designed transfer vector mutants, 293T cells were co-transfected with equal amounts of WT and mutant transfer vectors alongside the packaging plasmids to measure the RPE of all described U5–AUG mutants. RPE measurements revealed a two and a half fold and a fivefold reduction in the RPE of the U5s and LU5AUG mutants respectively (*n* = 8 for U5s, *P* = 0.000000008 and *n* = 3 for LU5AUG, *P* = 0.006, Figure 8C), while as shown above (Figure 2C) the S1 RPE did not differ from that of WT. Despite the established relationship between dimerization and packaging (26,48), these results suggest that dimerization enhancement does not automatically lead to increased packaging and suggest that structural flexibility of the RNA must be an important parameter for lentiviral vector packaging efficiency. Finally, to study the effect of these mutants on infectivity, we measured both physical and infectious titres of all viral vector supernatants by performing independent transfections for all transfer vector constructs as described above. As expected, graph 8D shows that the transduction efficiency of the mutant transfer vectors followed the exact same trend with their respective relative packaging efficiency with S1 having similar titers to WT (*n* = 12 for both WT and S1, *P* = 0.2 by an independent two sample *t* test with a two-tailed distribution), while U5s and LU5AUG were characterized by a twofold and a fourfold reduction in their functional titers (*n* = 12 for WT and *n* = 7 for U5s,*P* = 0.02, and *n* = 6 for LU5AUG, *P* = 0.0002,). Lastly, ELLA p24 was performed to measure physical vector titer (Figure 8E). WT, U5s, S1 and LU5AUG produced physical titers comparable to their infectious titres, suggesting that mutations in the U5–AUG motif of the 5′UTR did not influence Gag expression when provided *in trans* (aliquots from the same viral supernatant repeats were analysed both for physical and functional titres).

Overall, our data suggest that structural flexibility of the packaging signal region of the RNA is a prerequisite for efficient packaging and that sequences that allow for a structural equilibrium between the newly identified U5–*gag* and U5–AUG interactions enhance packaging. Destabilization of the U5–*gag* with parallel strengthening of the U5–AUG structural motif can lead to increased dimerization, but modifications that overstabilize or destabilize these interchangeable structures can be deleterious for RNA encapsidation despite increased dimerization properties.

## DISCUSSION

Lentiviral vectors are being successfully used as therapeutic agents in a series of clinical applications of gene therapy including genome editing and cancer immunotherapy. 3^rd^ generation HIV-1 derived lentiviral vectors are produced from 4 independent plasmids, which separate the components of the virus required for successful gene delivery (42). Entry, nuclear export of the vector RNA, translation of Gag and the signals for Gag to package the vector RNA are each governed by different plasmids, unlike in the native viral context, where one RNA performs all of these functions (42). We therefore aimed to improve the efficiency of vector genome packaging of LVs by specifically optimizing the dimeric RNA structure that promotes packaging at the expense of monomeric structures that are used by the virus for translational control. Although mutations predicted to enhance dimerization by stabilizing the U5–AUG helix produced a higher proportion of dimeric RNA, they did not enhance packaging, and instead revealed the necessity for structural flexibility in the U5–AUG in order to enable wild-type levels of packaging.

Lentiviral vectors have empirically included the 5′ region of the *gag* as an extended *psi* region although the mechanistic basis underlying this has previously been obscure. We have now identified long-range interactions between poly(A) and U5 sequences and the *gag* sequence, that are adopted by the dimeric RNA in preference to the widely published U5–AUG helix. The U5–AUG helix is an important component of the generally accepted structure of the optimal packaging signal in the 5′ UTR and for the virus it may be of advantage to restrict generation of a competent packaging structure in favour of a structure optimizing a translation phenotype for the full length RNA until the optimal conditions (e.g. adequate amounts of Gag and Gag/Pol polyprotein) are available. A mechanism in which structures that act as higher-affinity packaging signals on the gRNA do not form, might favour translation initially. However a population of the full length RNA has at some stage to become packageable and the availability of lower-affinity signals dependent on higher concentrations of Gag to trigger the full packaging process would be a logical strategy. This cooperative nature of packaging dependent on Gag concentration has previously been observed in cell culture (49).

The U5–AUG duplex has been proposed to form as an intermolecular interaction during dimer maturation (39, 50, 51).The presence of the U5–*gag* interaction may mean that the dimer never adopts an intramolecular U5–AUG duplex, but that, upon dimer maturation, in the presence of Gag, it switches from intermolecular U5–*gag* to an intermolecular U5–AUG conformation. Alternatively, competition between the U5–AUG and U5–*gag* structures in the immature dimer may destabilize the U5–AUG enough to remodel the initial dimer into a more extended intermolecular structure, including an intermolecular U5–AUG.

Our results suggest that sequences that allow for a structural equilibrium between the newly identified U5–*gag* and U5–AUG interactions lead to preferential packaging, whilst mutations that either overstabilize or destabilize these interchangeable structures can be deleterious for vector functionality despite increased dimerization properties. The observation that two of the mutants have an even greater impact on infectivity than upon packaging (Figure 8C,D) suggests that the regulation of these structures also impacts upon other stages of the viral lifecycle, such as, perhaps, reverse transcription (52). Our *in virio* and *in vitro* data support the presence and importance of an expanded structural equilibrium of the *cis-*acting elements of HIV-1 and shed light upon the role of the 5′ *gag* sequences for the maintenance of lentiviral vector titres. Additionally, this work confirms previous structural findings of this leader and *gag* region in the context of a WT HIV-1 structural analysis inside virions. Watts *et al.* showed previously that HIV-1 particles contain dimeric RNA that adopts the U5–AUG duplex 5′ of *gag* sequences that form independent stem loops in a similar manner to the BMH-type dimeric structure we show here (53). The *in virio* data detects the average stable dimeric structure inside virions, suggesting, as we do, that the U5–AUG interaction is the dominant structure inside mature virions and that it is likely to be important for the later stages of packaging and maturation. The lack of U5–*gag* and polyA–*gag* interactions seen within their study might reflect a distinct role for these motifs during the cellular stage of the viral lifecycle or its transient nature within the virion (or both). Given the evidence that this region is important in encapsidation (19–23) we favour the latter role and hence we are describing it as the metastable dimer.

These novel structural findings which were shown to be supported by both phylogenetic and functional assessments help to consolidate disparate observations in the lentiviral packaging field and to uncover functional reasons for extended packaging signal sequences within the *gag* gene. They raise the possibility of a stepwise thermodynamically favourable pathway beginning with a stable monomeric RNA and ending with formation of the mature genomic dimer. This improvement in our understanding of the structure of the vector RNA could be used to improve the basic design of the lentiviral vector backbone employed for clinical applications of gene therapy as well as to identify novel drug targets in the RNA genome. More structurally informed approaches to enhancing RNA packaging will now be feasible based on these results and may help to enhance vector efficiency.

## DATA AVAILABILITY

SHAPE data used for structural modelling are provided as Supplementary Table S1.

## Supplementary Material

gkab1206_Supplemental_File

## Notes

Present address: Eirini Vamva, Center for Immunity and Immunotherapies, Seattle Children's Research Institute, Seattle, WA 98101, USA.
