# Supplementary material for: A novel role for gag as a cis-acting element regulating RNA structure, dimerization and packaging in HIV-1 lentiviral vectors
Source: Nucleic Acids Res. 2021 Dec 20;50(1):430–48. doi: 10.1093/nar/gkab1206 (PMC8754630; doi:10.1093/nar/gkab1206)
Supplement: gkab1206_Supplemental_File [file gkab1206_supplemental_file.pdf]

Monomer input data for RNAstructure

seqnum	seq	WT M	WT+gag M	S1+gag M	U5s+gag M	LU5AUG+gag M
7	U	-999	-999	-999	-999	-999
8	C	-999	-999	-999	-999	-999
9	U	-999	-999	-999	-999	-999
10	G	-999	-999	-999	-999	-999
11	G	-999	-999	-999	-999	-999
12	U	-999	-999	-999	-999	-999
13	U	-999	-999	-999	-999	-999
14	A	-999	-999	-999	-999	-999
15	G	-999	-999	-999	-999	-999
16	A	-999	-999	-999	-999	-999
17	C	-999	-999	-999	-999	-999
18	C	-999	-999	-999	-999	-999
19	A	-999	-999	-999	-999	-999
20	G	-999	-999	-999	-999	-999
21	A	0.5702	0.6031	0.2627	-999	-999
22	U	0.6311	0.4593	0.3605	-999	-999
23	C	0.3188	0.4741	0.1656	-999	1.1585
24	U	0.3398	0.2343	0.311	-999	0.7153
25	G	0.0356	0.1213	0.0599	-999	0.4875
26	A	0.3017	0.1751	0.1826	-999	0.0865
27	G	0.0217	0.115	0.0241	-999	0.4001
28	C	0.0512	0.0958	-0.0011	0.6725	0.2269
29	C	0.62	0.7483	0.3784	-0.323	-0.107
30	U	0.2596	0.6284	0.2215	0.0447	0.1263
31	G	1.8543	1.49	2.5044	0.5191	0.7737
32	G	0.1604	0.7799	-0.1241	1.8292	0.5971
33	G	0.9491	0.9514	0.6936	0.2903	0.2645
34	A	1.2484	0.996	1.1158	-0.13	0.4992
35	G	-0.0419	0.1228	0.4901	0.4109	0.7577
36	C	0.0954	0.2615	0.3536	-0.04	0.0716
37	U	0.0406	-0.122	0.1761	0.0595	0.0921
38	C	0.01	0.1823	-0.0822	0.0227	0.1294
39	U	0.0367	-0.107	0.0274	0.0317	0.0579
40	C	-0.0119	0.0497	-0.032	0.021	0.005
41	U	0.2753	0.1542	0.5783	0.1026	0.0359
42	G	0.2002	0.2483	0.1684	0.0025	0.4462
43	G	0.1661	-0.014	0.0376	0.3012	0.2893
44	C	0.09	0.2562	-0.0527	0.408	0.3297
45	U	0.4609	0.2999	0.3262	0.534	0.2023
46	A	0.2918	0.2068	0.1575	0.124	0.2549
47	A	-0.0545	0.0231	-0.0039	0.3299	0.3847

**48**	**C**	0.0267	-0.106	0.0941	0.1516	0.1981
**49**	**U**	0.1051	-0.07	0.0393	0.0997	0.0098
**50**	**A**	0.1845	-0.096	0.6852	0.2056	-0.004
**51**	**G**	0.2573	-0.043	0.1633	0.2371	0.0134
**52**	**G**	0.0408	0.1917	0.4433	0.0449	0.3794
**53**	**G**	0.166	0.1778	0.2388	0.4526	0.4046
**54**	**A**	0.0041	0.451	1.0288	0.1844	0.2941
**55**	**A**	0.7589	0.2363	0.9593	-0.177	0.1302
**56**	**C**	0.0376	0.9114	0.272	0.0205	2.4806
**57**	**C**	0.567	0.6822	2.4849	-0.151	0.4678
**58**	**C**	1.0645	0.0582	0.7326	-0.168	0.9672
**59**	**A**	-0.0279	0.5868	1.1615	0.2389	1.9833
**60**	**C**	0.2204	0.8972	0.5346	-0.112	-0.053
**61**	**U**	1.412	1.2937	-0.0213	0.8976	0.0206
**62**	**G**	0.3412	0.9243	0.5617	0.1985	1.3038
**63**	**C**	0.4267	0.2521	0.1297	0.2158	0.3128
**64**	**U**	0.4008	1.492	0.3174	0.0221	-0.032
**65**	**U**	5.0374	4.9837	1.5475	2.0739	-0.514
**66**	**A**	0.9123	0.7609	-0.2439	0.213	0.1351
**67**	**A**	0.9883	0.823	0.8293	0.1584	0.0665
**68**	**G**	0.0078	0.0957	0.0624	0.3229	0.2422
**69**	**C**	0.1398	0.0232	0.103	0.124	0.1285
**70**	**C**	0.0564	0.0753	0.0129	0.1744	0.167
**71**	**U**	0.2697	-0.116	0.1601	0.1655	0.0171
**72**	**C**	0.6831	0.0868	0.1689	0.1056	-0.053
**73**	**A**	1.1746	1.1852	0.758	0.8461	0.2162
**74**	**A**	4.3118	2.3607	1.2558	1.5538	0.3231
**75**	**U**	1.4878	1.6052	0.8119	0.4893	0.5097
**76**	**A**	1.2978	0.5453	0.0005	0.0024	0.4036
**77**	**A**	0.7737	0.7318	0.7033	0.5404	0.4541
**78**	**A**	0.602	0.576	0.7042	0.483	0.5702
**79**	**G**	-0.0437	0.0938	0.2247	0.0512	0.2445
**80**	**C**	0.0216	0.0225	0.1363	0.3198	0.3827
**81**	**U**	0.1211	0.3663	0.0215	0.0445	0.009
**82**	**U**	0.4087	-0.086	0.6628	-0.203	0.1645
**83**	**G**	0.361	0.1971	0.1393	0.3782	2.1558
**84**	**C**	0.0984	0.065	0.1427	0.118	0.1953
**85**	**C**	0.1703	0.1464	-0.0084	0.2524	0.3068
**86**	**U**	-0.0296	0.1722	0.0717	-0.01	0.1384
**87**	**U**	0.2318	0.1479	0.1138	0.0514	-0.073
**88**	**G**	0.155	0.2773	0.2237	0.0515	0.2156
**89**	**A**	0.0728	0.1809	0.1247	0.011	0.11
**90**	**G**	0.1761	0.1632	0.2376	0.1252	0.1714

**91**	**U**	0.1676	0.2511	0.0434	0.0272	0.0277
**92**	**G**	0.1438	0.0228	0.1909	0.2122	0.1876
**93**	**C**	0.0518	0.1287	0.0602	0.1973	0.0907
**94**	**U**	0.0441	0.0509	0.082	0.3526	0.3617
**95**	**U**	-0.0103	-0.185	0.0973	0.0956	0.0156
**96**	**C**	0.6723	0.1965	0.0368	0.635	0.0057
**97**	**A**	0.6202	0.3839	0.3394	0.488	0.3083
**98**	**A**	0.1112	0.2705	0.463	-0.029	0.2983
**99**	**G**	0.8096	0.2312	0.2352	0.5229	0.3118
**100**	**U**	1.5909	0.5822	0.2191	0.5572	-0.107
**101**	**A**	0.5102	0.4244	1.4144	0.4952	-0.172
**102**	**G**	0.1251	0.2426	0.4798	0.6872	0.9435
**103**	**U**	0.1488	0.4094	0.7015	0.6574	0.651
**104**	**G**	0.2218	0.3104	0.3126	0.1787	0.2098
**105**	**U**	0.0834	0.5189	-0.095	0.0881	0.5715
**106**	**G**	0.3957	0.4713	-0.1567	-0.063	0.284
**107**	**U**	0.8667	0.7867	0.1801	0.2584	0.2225
**108**	**G**	0.1515	0.1053	0.256	0.2326	0.2557
**109**	**C**	0.0768	0.084	-0.0007	0.2457	0.2185
**110**	**C**	0.003	0.1168	0.1189	0.1575	0.2439
**111**	**C**	0.2489	0.1716	0.2408	0.1065	-0.016
**112**	**G**	0.0146	0.1963	0.1399	0.0626	0.2089
**113**	**U**	0.188	0.0977	0.2425	0.1275	0.2296
**114**	**C**	0.1839	0.226	0.3662	0.3391	0.4399
**115**	**U**	0.3293	0.5429	0.2734	0.3063	0.1572
**116**	**G**	0.3163	0.4134	0.6522	-0.099	0.058
**117**	**U**	0.555	0.4909	1.0043	1.4198	0.472
**118**	**U**	0.8636	1.078	3.7548	2.1512	1.3433
**119**	**G**	-0.0032	0.3291	3.8551	6.2987	1.6688
**120**	**U**	0.6054	0.3862	4.9277	3.5896	0.4152
**121**	**G**	0.059	0.522	-0.8377	-1.317	-0.373
**122**	**U**	0.5542	0.2726	9.1644	1.7588	1.7877
**123**	**G**	0.079	0.0837	1.2758	0.8457	0.8082
**124**	**A**	0.043	0.1921	0.6413	1.0711	1.212
**125**	**C**	0.0427	0.0513	-0.1398	1.0528	1.2909
**126**	**U**	0.0117	0.1384	0.3196	0.5996	0.6634
**127**	**C**	0.123	0.1092	0.0977	0.2534	0.3184
**128**	**U**	0.1915	0.1143	0.0418	0.1414	0.1855
**129**	**G**	0.1205	0.0304	0.3546	0.044	0.219
**130**	**G**	-0.0449	0.0951	-0.6512	0.0064	0.1743
**131**	**U**	1.1755	0.3738	0.7399	0.6492	-0.164
**132**	**A**	0.607	0.5126	0.3441	0.394	-0.071
**133**	**A**	0.1868	0.1332	0.2018	0.2873	0.5891

**134**	**C**	0.3589	0.1235	0.4036	0.1443	0.1545
**135**	**U**	0.3196	0.1991	0.0986	0.0057	0.0335
**136**	**A**	0.259	0.4671	0.1687	0.3536	-0.225
**137**	**G**	0.0948	0.0807	0.1203	0.1287	0.2791
**138**	**A**	0.1266	0.0408	0.3053	0.0118	0.0924
**139**	**G**	0.0973	0.2484	0.1931	0.2836	0.3544
**140**	**A**	0.0147	0.0846	0.1254	-0.04	0.0125
**141**	**U**	0.3677	0.3677	0.4122	1.453	1.6789
**142**	**C**	0.1951	0.2932	0.2853	0.4128	2.533
**143**	**C**	0.47	0.1475	-0.0044	0.1627	0.4028
**144**	**C**	0.0599	0.049	0.0729	0.1019	0.1882
**145**	**U**	0.4606	0.3676	0.0925	0.3285	0.1383
**146**	**C**	0.7133	0.3179	0.0284	-0.174	-0.131
**147**	**A**	1.5139	0.6891	0.2786	0.5925	-0.097
**148**	**G**	-0.2644	-0.042	-0.1825	0.0268	0.0015
**149**	**A**	3.3618	1.6543	-0.3286	3.1308	0.2736
**150**	**C**	1.6261	1.0651	0.183	-0.145	0.5084
**151**	**C**	1.0636	0.6865	0.1852	-0.308	1.0529
**152**	**C**	0.9185	0.9696	0.6389	1.0787	2.0842
**153**	**U**	0.4773	0.7122	0.2971	0.4271	2.4803
**154**	**U**	0.0539	0.1577	0.1064	0.1135	0.3906
**155**	**U**	1.3147	1.7164	0.9647	1.1434	0.9819
**156**	**U**	2.7045	2.4705	0.4124	0.7316	1.0472
**157**	**A**	1.1249	0.6445	1.0893	0.5476	0.7572
**158**	**G**	0.1045	0.0518	0.4158	0.1747	0.5408
**159**	**U**	0.2921	0.2504	0.2688	0.2644	-0.013
**160**	**C**	0.3344	0.0072	0.2326	0.0739	-0.023
**161**	**A**	0.4059	0.4812	0.5316	0.5481	0.0535
**162**	**G**	0.1905	0.2181	0.1157	0.2292	0.2555
**163**	**U**	0.0656	-0.229	0.0484	-0.054	0.0539
**164**	**G**	0.4497	0.6645	0.3351	0.2227	0.2594
**165**	**U**	1.1543	0.5278	1.261	1.1292	1.0042
**166**	**G**	-0.0089	0.5119	0.5329	0.4691	0.9045
**167**	**G**	0.1803	0.6213	0.2977	0.4077	0.4052
**168**	**A**	0.6748	1.035	0.775	0.1975	0.0118
**169**	**A**	0.001	0.0573	0.2094	0.6896	0.5246
**170**	**A**	1.3052	1.3572	0.8443	0.4014	0.2711
**171**	**A**	0.2303	0.4481	0.1628	0.022	0.6148
**172**	**U**	0.1672	0.2007	0.4396	0.1351	0.2847
**173**	**C**	0.0388	-0.022	0.1387	0.0478	0.115
**174**	**U**	0.0288	-0.045	0.165	0.0916	0.0679
**175**	**C**	0.1871	0.1885	0.1046	0.1278	0.0931
**176**	**U**	1.4166	0.5929	0.4063	0.146	0.0182

**177**	**A**	0.61	0.4322	0.2202	0.7066	-0.144
**178**	**G**	1.5695	0.1422	0.5064	0.4504	0.4223
**179**	**C**	3.1363	-0.488	0.6887	0.4356	0.164
**180**	**A**	1.0334	0.5489	0.3955	0.2741	-0.244
**181**	**G**	0.3297	0.0826	0.4716	0.1336	0.241
**182**	**U**	0.2737	0.2068	0.7863	0.1108	0.0117
**183**	**G**	0.0359	-0.049	0.8658	-0.419	0.2075
**184**	**G**	0.1459	0.3412	0.3734	1.3577	0.144
**185**	**C**	0.2387	0.3837	0.3336	0.2312	0.9221
**186**	**G**	0.2069	0.2948	0.3025	0.0847	0.2946
**187**	**C**	0.0195	0.0427	0.1035	-0.022	0.1176
**188**	**C**	0.0875	0.1212	0.1129	0.0585	0.0775
**189**	**C**	0.2287	0.3741	0.0031	-0.022	0.2395
**190**	**G**	0.5228	0.4878	0.2596	0.2166	0.1153
**191**	**A**	1.2271	1.501	0.4404	0.6273	0.2254
**192**	**A**	2.1155	1.3784	0.0445	1.3541	0.3809
**193**	**C**	0.8505	0.914	0.3954	-0.708	0.2768
**194**	**A**	0.1944	0.6207	0.4227	0.3456	0.3049
**195**	**G**	0.0112	-0.075	0.1828	-0.055	0.1537
**196**	**G**	0.0063	-0.01	0.2766	0.0149	-0.246
**197**	**G**	0.3465	0.6802	0.0625	0.3923	1.196
**198**	**A**	0.1709	0.3666	0.0694	0.2972	1.0984
**199**	**C**	-0.0154	-0.056	0.1757	0.356	0.2353
**200**	**C**	0.3533	0.1952	0.4586	0.8918	0.1467
**201**	**U**	0.7544	0.5015	0.5442	1.9916	0.7137
**202**	**G**	0.3354	0.379	0.684	0.3197	0.4698
**203**	**A**	0.1069	0.1954	0.603	0.0794	0.1914
**204**	**A**	0.2912	0.3267	0.5435	0.2608	0.3462
**205**	**A**	0.3764	0.4531	0.428	0.8831	0.5903
**206**	**G**	0.2639	0.2578	0.1717	0.1695	0.3423
**207**	**C**	1.3929	0.6003	0.2913	0.5296	0.1128
**208**	**G**	0.0656	0.2414	0.0983	-0.123	-0.088
**209**	**A**	0.3522	0.4792	0.3749	0.3732	0.1556
**210**	**A**	0.2889	0.6167	0.1665	0.0641	0.2792
**211**	**A**	0.2646	0.6948	0.4616	0.427	0.2002
**212**	**G**	0.5183	0.6059	-0.0123	0.4963	0.2177
**213**	**G**	0.5309	0.04	0.367	0.0513	0.6518
**214**	**G**	0.2611	0.5274	0.4464	-0.017	-0.136
**215**	**A**	0.4378	0.5622	0.2803	0.8137	0.5912
**216**	**A**	0.475	0.557	0.4164	0.1853	0.4687
**217**	**A**	0.158	0.1761	0.1006	0.09	0.5561
**218**	**C**	0.0711	-0.082	0.1831	0.0852	0.2817
**219**	**C**	0.5104	0.1629	0.3096	0.4196	-0.084

**220**	**A**	0.1541	0.2401	0.4035	-0.017	-0.162
**221**	**G**	-0.0056	0.0873	0.0866	0.0878	0.1548
**222**	**A**	0.0742	0.3182	0.2914	0.0358	0.0091
**223**	**G**	0.0068	0.1033	0.2294	-0.037	0.181
**224**	**G**	0.2981	0.2821	0.281	0.4099	0.1575
**225**	**A**	0.2527	0.2747	0.2147	-0.05	0.2531
**226**	**G**	0.1594	0.2108	0.3941	0.3527	0.286
**227**	**C**	0.0356	0.0837	0.081	0.0767	0.4154
**228**	**U**	0.0933	0.1278	0.2434	0.2236	0.3781
**229**	**C**	0.0294	0.0662	0.1307	-0.021	0.2446
**230**	**U**	0.5859	0.2066	0.2219	0.0606	0.1176
**231**	**C**	1.0752	0.7087	0.2343	0.2769	0.2246
**232**	**U**	0.2254	0.0995	0.1076	0.0231	0.1828
**233**	**C**	0.3268	0.254	0.4019	0.3381	0.019
**234**	**G**	0.0132	0.0312	0.141	0.1741	0.2722
**235**	**A**	0.1828	0.3194	0.4776	1.065	0.7661
**236**	**C**	0.3305	0.1891	0.3706	0.2475	0.7452
**237**	**G**	0.5851	0.2761	0.1307	0.1601	0.5097
**238**	**C**	6.0104	2.478	0.5165	1.4194	0.0532
**239**	**A**	0.8329	0.5149	0.1781	0.3511	0.838
**240**	**G**	0.5823	0.4667	1.5445	1.7417	0.5244
**241**	**G**	0.3198	0.2731	-0.0719	-0.077	1.1003
**242**	**A**	0.3265	0.4788	0.838	-0.016	0.7068
**243**	**C**	0.1947	0.2887	0.2659	0.1132	0.2847
**244**	**U**	0.0629	0.1676	0.1498	-0.054	0.1324
**245**	**C**	1.3265	1.3643	0.5104	0.1671	0.0998
**246**	**G**	0.4089	0.5167	0.2578	1.1358	1.3797
**247**	**G**	0.2521	0.3332	0.2923	0.066	0.3306
**248**	**C**	0.195	0.1363	-0.2166	0.1668	0.3495
**249**	**U**	0.9403	0.5545	0.5695	0.3215	0.1036
**250**	**U**	0.8467	0.7319	0.7269	0.5786	0.2783
**251**	**G**	0.0023	0.7381	0.0756	0.1402	0.3942
**252**	**C**	0.2648	0.1752	0.1987	0.147	0.5124
**253**	**U**	0.26	0.2909	0.2881	0.1212	0.0757
**254**	**G**	0.177	0.2468	-0.0524	-0.109	0.2621
**255**	**A**	0.3458	0.4008	0.1703	0.4784	0.4526
**256**	**A**	0.2704	0.1827	-0.0744	-0.002	0.1637
**257**	**G**	-0.1341	0.2674	0.5336	0.2983	0.1268
**258**	**C**	-0.0007	0.2527	0.1302	0.0447	0.2149
**259**	**G**	0.056	0.2738	0.6257	-0.004	-0.149
**260**	**C**	0.1028	0.0367	0.4832	-0.408	0.3996
**261**	**G**	0.1211	-0.018	0.173	0.1824	0.2166
**262**	**C**	0.3752	-0.037	0.2564	0.2726	-0.059

**263**	**A**	0.0854	0.1725	0.1814	0.0705	0.1375
**264**	**C**	0.4007	0.264	0.3788	0.4324	0.4263
**265**	**G**	0.1253	0.3402	0.3191	0.2947	0.4018
**266**	**G**	0.186	-0.075	0.0961	0.1198	0.0811
**267**	**C**	0.379	-0.051	0.5246	0.248	-0.21
**268**	**A**	0.0511	0.0304	0.0363	0.0515	0.0876
**269**	**A**	0.0611	0.1605	0.4277	0.143	0.06
**270**	**G**	0.0652	0.1513	-0.0794	-0.047	0.2829
**271**	**A**	0.4197	0.4736	0.2419	0.4512	0.2501
**272**	**G**	1.2238	0.8649	0.3689	-0.064	0.2967
**273**	**G**	2.1532	0.6984	0.2578	0.6669	0.8197
**274**	**C**	2.0042	0.7847	0.4452	0.4817	0.3702
**275**	**G**	0.2989	0.4234	0.4038	0.1886	0.0118
**276**	**A**	0.2687	0.2344	0.6014	-0.071	0.174
**277**	**G**	0.1489	0.0831	0.1966	0.1887	0.4912
**278**	**G**	-0.0234	-0.013	0.3473	-0.15	0.4623
**279**	**G**	0.123	0.1407	0.1055	0.0008	0.137
**280**	**G**	0.1363	0.3057	0.1478	0.1086	0.3074
**281**	**C**	-0.0951	0.1388	0.0754	-0.061	0.1299
**282**	**G**	0.7328	0.3078	-0.0718	0.3367	0.3346
**283**	**G**	0.8522	0.1081	0.1441	0.0143	0.2945
**284**	**C**	1.3451	0.4293	0.3931	0.0881	0.0081
**285**	**G**	0.4395	0.5358	0.3627	0.1096	0.1446
**286**	**A**	0.2515	0.2074	0.1969	0.084	0.1716
**287**	**C**	0.1288	0.2026	0.1681	0.2125	0.2014
**288**	**U**	0.476	0.8081	0.474	0.4879	0.5046
**289**	**G**	0.5457	0.764	-0.5128	0.3922	0.1806
**290**	**G**	0.2623	0.897	0.2982	0.6507	0.3616
**291**	**U**	1.0471	0.2708	0.7005	1.7992	0.9225
**292**	**G**	1.4629	2.0102	0.93	0.1916	0.3435
**293**	**A**	0.0827	0.7304	0.2019	0.1383	1.3682
**294**	**G**	0.5718	0.7975	0.6092	0.863	0.0271
**295**	**U**	1.9608	1.9645	0.7332	0.5046	0.1277
**296**	**A**	0.3998	1.1669	0.7298	0.0627	-0.183
**297**	**C**	0.0996	0.253	0.3651	0.0441	1.1839
**298**	**G**	1.0799	0.0727	0.5391	0.7803	0.9974
**299**	**C**	0.4242	0.1572	-0.0051	0.5308	0.4852
**300**	**C**	0.7649	0.4618	0.4168	1.0279	0.9808
**301**	**A**	0.5399	0.2905	0.4888	1.0042	1.0552
**302**	**A**	0.2507	0.2139	0.2438	0.2181	0.6532
**303**	**A**	0.2457	0.3393	0.2916	0.5787	1.0757
**304**	**A**	0.6197	0.3255	0.2965	1.452	1.3411
**305**	**A**	0.5366	0.2665	0.5595	1.5255	0.9044

**306**	**U**	0.1033	0.1609	0.2275	0.0554	0.7599
**307**	**U**	0.3029	0.1935	0.4608	0.3058	0.3039
**308**	**U**	0.2323	0.4763	0.2366	0.2259	0.5701
**309**	**U**	1.7807	0.4767	1.5019	1.5654	0.3412
**310**	**G**	0.6687	0.3796	0.8871	1.1236	0.7883
**311**	**A**	0.3386	0.531	0.9516	0.3636	0.4132
**312**	**C**	0.1405	0.1378	0.2259	0.0451	0.4921
**313**	**U**	0.5477	0.5039	0.0745	0.5006	0.0461
**314**	**A**	0.2492	0.1598	0.6177	0.0234	-0.306
**315**	**G**	0.1306	0.0076	0.2003	0.2088	0.398
**316**	**C**	0.0348	-0.059	0.1954	0.1227	0.0773
**317**	**G**	0.1814	0.1605	0.4893	-0.046	-0.099
**318**	**G**	0.89	0.4955	0.5296	0.7037	0.1721
**319**	**A**	1.9677	0.7511	0.5927	3.0104	0.6255
**320**	**G**	1.7756	0.5749	1.5869	0.9181	2.5337
**321**	**G**	0.1842	-0.05	0.3096	0.0207	0.9274
**322**	**C**	0.1446	0.005	0.3923	0.2345	0.259
**323**	**U**	0.4991	0.2459	0.3757	0.3494	0.0979
**324**	**A**	0.0581	0.1421	0.3412	0.0583	-0.193
**325**	**G**	0.3027	0.1381	0.1913	0.3641	0.0847
**326**	**A**	0.4334	0.1467	0.5068	0.4273	0.2799
**327**	**A**	0.1948	0.1247	0.3252	0.1164	0.3885
**328**	**G**	0.2705	0.0869	0.6338	-0.16	0.2773
**329**	**G**	0.1733	0.2122	0.1355	0.08	0.6261
**330**	**A**	0.0756	0.1126	0.1881	0.1155	0.1728
**331**	**G**	0.0386	0.1543	0.0692	0.3928	0.2485
**332**	**A**	0.6932	0.4734	-0.2139	0.0964	0.0879
**333**	**G**	0.272	0.3804	0.9408	0.4205	0.8178
**334**	**A**	0.1439	0.369	0.0995	0.1159	0.1995
**335**	**G**	0.3157	0.3464	0.1767	0.1317	0.4173
**336**	**A**	0.1393	0.0807	0.1042	0.0591	0.1157
**337**	**U**	0.9826	0.5029	0.0557	0.1103	0.0946
**338**	**G**	0.6303	0.346	0.5348	0.0058	0.3057
**339**	**G**	0.0715	0.019	0.7052	0.0241	0.6123
**340**	**G**	0.4868	0.2785	0.0482	0.0469	0.3005
**341**	**U**	0.7847	0.1076	0.1457	0.3224	0.4249
**342**	**G**	0.1873	0.2337	0.2542	0.0171	0.3969
**343**	**C**	0.1141	0.5429	0.1972	0.4164	0.0321
**344**	**G**	0.8776	0.365	0.497	1.477	2.1766
**345**	**A**	0.0831	0.0961	0.9101	0.0571	2.0947
**346**	**G**	0.4915	0.3093	0.5479	0.5875	-0.374
**347**	**A**	0.4452	0.6153	1.2676	4.3461	0.6679
**348**	**G**	1.3278	-0.026	1.3923	0.1249	0.4059

**349**	**C**	0.2923	0.073	0.2924	0.6137	5.6716
**350**	**G**	0.0622	0.0323	0.3025	0.1217	0.3327
**351**	**U**	0.2583	-0.306	0.9382	0.9141	0.0656
**352**	**C**	1.5481	-0.694	0.7199	0.1032	0.3634
**353**	**A**		1.14	2.587	0.5047	0.4826
**354**	**G**		1.3793	2.5464	0.5065	4.1454
**355**	**U**		1.6851	-0.1501	3.9542	1.2889
**356**	**A**		0.1503	6.3889	0.1207	-0.027
**357**	**U**		0.9647	-0.3365	1.4838	0.8082
**358**	**U**		2.0192	0.1369	2.166	-0.611
**359**	**A**		-0.682	-8.9011	0.2766	0.3396
**360**	**A**		0.3662	35.043	-1.078	-0.428
**361**	**G**		0.0446	0.3005	-999	-2.576
**362**	**C**		0.2042	11.273	-999	2.6614
**363**	**G**		0.0931	0.1805	-999	-999
**364**	**G**		-0.307	-999	-999	-999
**365**	**G**		0.7882	-999	-999	-999
**366**	**G**		1.4685	-999	-999	-999
**367**	**G**		1.3704	-999	-999	-999
**368**	**A**		0.7733	-999	-999	-999
**369**	**G**		0.1543	-999	-999	-999
**370**	**A**		0.3575	-999	-999	-999
**371**	**A**		0.2335	-999	-999	-999
**372**	**U**		0.4315	-999	-999	-999
**373**	**U**		1.8849	-999	-999	-999
**374**	**A**		0.7101	-999	-999	-999
**375**	**G**		-0.291	-999	-999	-999
**376**	**A**		-0.161	-999	-999	-999
**377**	**U**		0.4825	-999	-999	-999
**378**	**C**		0.1666	-999	-999	-999
**379**	**G**		0.1678	-999	-999	-999
**380**	**C**		0.4851	-999	-999	-999
**381**	**G**		0.2626	-999	-999	-999
**382**	**A**		0.0396	-999	-999	-999
**383**	**U**		-0.093	-999	-999	-999
**384**	**G**		0.564	-999	-999	-999
**385**	**G**		1.4006	-999	-999	-999
**386**	**G**		1.6721	-999	-999	-999
**387**	**A**		0.5097	-999	-999	-999
**388**	**A**		0.1354	-999	-999	-999
**389**	**A**		0.0881	-999	-999	-999
**390**	**A**		0.5388	-999	-999	-999
**391**	**A**		0.1491	-999	-999	-999

**392**	**A**	0.3805	-999	-999	-999
**393**	**U**	0.2371	-999	-999	-999
**394**	**U**	0.4889	-999	-999	-999
**395**	**C**	0.665	-999	-999	-999
**396**	**G**	0.5319	-999	-999	-999
**397**	**G**	0.5689	-999	-999	-999
**398**	**U**	-0.021	-999	-999	-999
**399**	**U**	0.3662	-999	-999	-999
**400**	**A**	-0.058	-999	-999	-999
**401**	**A**	0.2494	-999	-999	-999
**402**	**G**	0.1079	-999	-999	-999
**403**	**G**	-0.047	-999	-999	-999
**404**	**C**	-0.077	-999	-999	-999
**405**	**C**	-0.005	-999	-999	-999
**406**	**A**	0.0877	-999	-999	-999
**407**	**G**	0.1547	-999	-999	-999
**408**	**G**	0.2441	-999	-999	-999
**409**	**G**	0.2236	-999	-999	-999
**410**	**G**	1.3812	-999	-999	-999
**411**	**G**	0.2116	-999	-999	-999
**412**	**A**	0.4378	-999	-999	-999
**413**	**A**	0.2428	-999	-999	-999
**414**	**A**	0.5086	-999	-999	-999
**415**	**G**	0.2031	-999	-999	-999
**416**	**A**	0.3444	-999	-999	-999
**417**	**A**	0.2802	-999	-999	-999
**418**	**A**	0.1865	-999	-999	-999
**419**	**A**	0.1814	-999	-999	-999
**420**	**A**	0.2812	-999	-999	-999
**421**	**A**	0.7501	-999	-999	-999
**422**	**U**	0.7187	-999	-999	-999
**423**	**A**	-0.15	-999	-999	-999
**424**	**U**	2.0177	-999	-999	-999
**425**	**A**	0.2282	-999	-999	-999
**426**	**A**	0.2112	-999	-999	-999
**427**	**A**	0.3753	-999	-999	-999
**428**	**U**	0.5606	-999	-999	-999
**429**	**U**	1.4716	-999	-999	-999
**430**	**A**	0.485	-999	-999	-999
**431**	**A**	0.0914	-999	-999	-999
**432**	**A**	0.0647	-999	-999	-999
**433**	**A**	0.2615	-999	-999	-999
**434**	**C**	0.5454	-999	-999	-999

**435**	**A**	0.32	-999	-999	-999
**436**	**U**	0.6724	-999	-999	-999
**437**	**A**	-0.243	-999	-999	-999
**438**	**U**	1.538	-999	-999	-999
**439**	**A**	0.1059	-999	-999	-999
**440**	**G**	0.4833	-999	-999	-999
**441**	**U**	1.1503	-999	-999	-999
**442**	**A**	-0.203	-999	-999	-999
**443**	**U**	1.7086	-999	-999	-999
**444**	**G**	0.027	-999	-999	-999
**445**	**G**	0.1202	-999	-999	-999
**446**	**G**	0.4221	-999	-999	-999
**447**	**C**	-0.235	-999	-999	-999
**448**	**A**	0.1649	-999	-999	-999
**449**	**A**	0.2254	-999	-999	-999
**450**	**G**	0.0991	-999	-999	-999
**451**	**C**	0.215	-999	-999	-999
**452**	**A**	0.3364	-999	-999	-999
**453**	**G**	0.3649	-999	-999	-999
**454**	**G**	0.3722	-999	-999	-999
**455**	**G**	4.03	-999	-999	-999
**456**	**A**	1.3261	-999	-999	-999
**457**	**G**	0.1194	-999	-999	-999
**458**	**C**	0.3079	-999	-999	-999
**459**	**U**	0.4481	-999	-999	-999
**460**	**A**	0.4351	-999	-999	-999
**461**	**G**	0.4268	-999	-999	-999
**462**	**A**	0.0735	-999	-999	-999
**463**	**A**	0.0578	-999	-999	-999
**464**	**C**	0.5277	-999	-999	-999
**465**	**G**	0.0079	-999	-999	-999
**466**	**A**	0.3468	-999	-999	-999
**467**	**U**	-0.088	-999	-999	-999
**468**	**U**	-0.014	-999	-999	-999
**469**	**C**	0.2705	-999	-999	-999
**470**	**G**	0.1764	-999	-999	-999
**471**	**C**	1.2787	-999	-999	-999
**472**	**A**	0.2573	-999	-999	-999
**473**	**G**	0.1684	-999	-999	-999
**474**	**U**	0.1344	-999	-999	-999
**475**	**U**	0.5056	-999	-999	-999
**476**	**A**	0.3795	-999	-999	-999
**477**	**A**	0.3835	-999	-999	-999

**478**	**U**	0.3293	-999	-999	-999
**479**	**C**	-0.085	-999	-999	-999
**480**	**C**	0.0584	-999	-999	-999
**481**	**U**	-0.185	-999	-999	-999
**482**	**G**	-0.237	-999	-999	-999
**483**	**G**	-0.072	-999	-999	-999
**484**	**C**	0.9005	-999	-999	-999
**485**	**C**	4.9726	-999	-999	-999
**486**	**U**	0.4487	-999	-999	-999
**487**	**G**	0.3351	-999	-999	-999
**488**	**U**	0.4483	-999	-999	-999
**489**	**U**	0.2534	-999	-999	-999
**490**	**A**	0.2977	-999	-999	-999
**491**	**G**	1.3508	-999	-999	-999
**492**	**A**	-0.032	-999	-999	-999
**493**	**A**	0.0985	-999	-999	-999
**494**	**A**	0.1619	-999	-999	-999
**495**	**C**	0.2903	-999	-999	-999
**496**	**A**	0.0799	-999	-999	-999
**497**	**U**	0.2265	-999	-999	-999
**498**	**C**	0.0476	-999	-999	-999
**499**	**A**	0.7525	-999	-999	-999
**500**	**G**	0.0279	-999	-999	-999
**501**	**A**	0.2397	-999	-999	-999
**502**	**A**	0.3736	-999	-999	-999
**503**	**G**	0.3194	-999	-999	-999
**504**	**G**	0.0929	-999	-999	-999
**505**	**C**	0.054	-999	-999	-999
**506**	**U**	0.1242	-999	-999	-999
**507**	**G**	0.0106	-999	-999	-999
**508**	**U**	0.5478	-999	-999	-999
**509**	**A**	0.1537	-999	-999	-999
**510**	**G**	0.1787	-999	-999	-999
**511**	**A**	0.633	-999	-999	-999
**512**	**C**	0.0789	-999	-999	-999
**513**	**A**	0.4062	-999	-999	-999
**514**	**A**	0.0399	-999	-999	-999
**515**	**A**	0.4842	-999	-999	-999
**516**	**U**	0.5079	-999	-999	-999
**517**	**A**	-0.186	-999	-999	-999
**518**	**C**	-0.122	-999	-999	-999
**519**	**U**	0.807	-999	-999	-999
**520**	**G**	0.0584	-999	-999	-999

**521**	**G**	-0.09	-999	-999	-999
**522**	**G**	1.4958	-999	-999	-999
**523**	**A**	-0.079	-999	-999	-999
**524**	**C**	-0.036	-999	-999	-999
**525**	**A**	0.3566	-999	-999	-999
**526**	**G**	-0.136	-999	-999	-999
**527**	**C**	0.0483	-999	-999	-999
**528**	**U**	0.1115	-999	-999	-999
**529**	**A**	0.091	-999	-999	-999
**530**	**C**	-0.307	-999	-999	-999
**531**	**A**	0.1658	-999	-999	-999
**532**	**A**	0.2612	-999	-999	-999
**533**	**C**	0.0549	-999	-999	-999
**534**	**C**	0.3662	-999	-999	-999
**535**	**A**	0.1569	-999	-999	-999
**536**	**U**	0.3728	-999	-999	-999
**537**	**C**	0.7041	-999	-999	-999
**538**	**C**	0.0876	-999	-999	-999
**539**	**C**	0.246	-999	-999	-999
**540**	**U**	0.0396	-999	-999	-999
**541**	**U**	0.3466	-999	-999	-999
**542**	**C**	0.3235	-999	-999	-999
**543**	**A**	0.2012	-999	-999	-999
**544**	**G**	0.0527	-999	-999	-999
**545**	**A**	-0.361	-999	-999	-999
**546**	**C**	0.6172	-999	-999	-999
**547**	**A**	0.2223	-999	-999	-999
**548**	**G**	0.412	-999	-999	-999
**549**	**G**	0.0075	-999	-999	-999
**550**	**A**	0.2839	-999	-999	-999
**551**	**U**	0.2683	-999	-999	-999
**552**	**C**	0.5643	-999	-999	-999
**553**	**A**	-0.042	-999	-999	-999
**554**	**G**	0.002	-999	-999	-999
**555**	**A**	0.2787	-999	-999	-999
**556**	**A**	0.1289	-999	-999	-999
**557**	**G**	0.0778	-999	-999	-999
**558**	**A**	0.2402	-999	-999	-999
**559**	**A**	0.2938	-999	-999	-999
**560**	**C**	0.193	-999	-999	-999
**561**	**U**	0.1269	-999	-999	-999
**562**	**U**	0.8957	-999	-999	-999
**563**	**A**	0.1771	-999	-999	-999

**564**	**G**	0.0886	-999	-999	-999
**565**	**A**	0.2444	-999	-999	-999
**566**	**U**	0.1828	-999	-999	-999
**567**	**C**	0.8256	-999	-999	-999
**568**	**A**	-0.016	-999	-999	-999
**569**	**U**	0.4782	-999	-999	-999
**570**	**U**	0.5797	-999	-999	-999
**571**	**A**	0.0678	-999	-999	-999
**572**	**U**	0.7249	-999	-999	-999
**573**	**A**	-0.419	-999	-999	-999
**574**	**U**	1.1421	-999	-999	-999
**575**	**A**	0.1305	-999	-999	-999
**576**	**A**	-0.106	-999	-999	-999
**577**	**U**	0.499	-999	-999	-999
**578**	**A**	-0.56	-999	-999	-999
**579**	**C**	-0.017	-999	-999	-999
**580**	**A**	-0.031	-999	-999	-999
**581**	**G**	0.9145	-999	-999	-999
**582**	**U**	0.492	-999	-999	-999
**583**	**A**	0.2924	-999	-999	-999
**584**	**G**	0.0711	-999	-999	-999
**585**	**C**	-0.07	-999	-999	-999
**586**	**A**	0.403	-999	-999	-999
**587**	**A**	4.2204	-999	-999	-999
**588**	**C**	0.2086	-999	-999	-999
**589**	**C**	0.0024	-999	-999	-999
**590**	**C**	-0.677	-999	-999	-999
**591**	**U**	-0.839	-999	-999	-999
**592**	**C**	-1.891	-999	-999	-999
**593**	**U**	-0.194	-999	-999	-999
**594**	**A**	0.264	-999	-999	-999
**595**	**U**	0.0337	-999	-999	-999
**596**	**U**	0.09	-999	-999	-999
**597**	**G**	0.4294	-999	-999	-999
**598**	**U**	-0.176	-999	-999	-999
**599**	**G**	2.1487	-999	-999	-999
**600**	**U**	0.717	-999	-999	-999
**601**	**G**	0.4007	-999	-999	-999
**602**	**C**	0.5032	-999	-999	-999
**603**	**A**	-0.182	-999	-999	-999
**604**	**U**	1.1253	-999	-999	-999
**605**	**C**	0.4388	-999	-999	-999
**606**	**A**	-0.066	-999	-999	-999

**607**	**A**	0.0637	-999	-999	-999
**608**	**A**	0.1143	-999	-999	-999
**609**	**G**	0.4178	-999	-999	-999
**610**	**G**	0.2345	-999	-999	-999
**611**	**A**	0.0892	-999	-999	-999
**612**	**U**	1.0013	-999	-999	-999
**613**	**A**	0.6601	-999	-999	-999
**614**	**G**	0.2872	-999	-999	-999
**615**	**A**	0.1555	-999	-999	-999
**616**	**G**	0.5611	-999	-999	-999
**617**	**A**	0.8317	-999	-999	-999
**618**	**U**	-0.641	-999	-999	-999
**619**	**A**	0.7414	-999	-999	-999
**620**	**A**	0.0809	-999	-999	-999
**621**	**A**	-0.052	-999	-999	-999
**622**	**A**	0.2781	-999	-999	-999
**623**	**G**	0.7011	-999	-999	-999
**624**	**A**	0.333	-999	-999	-999
**625**	**C**	0.4485	-999	-999	-999
**626**	**A**	0.2226	-999	-999	-999
**627**	**C**	0.2563	-999	-999	-999
**628**	**C**	0.2923	-999	-999	-999
**629**	**A**	0.3009	-999	-999	-999
**630**	**A**	0.5686	-999	-999	-999
**631**	**G**	0.7484	-999	-999	-999
**632**	**G**	0.4904	-999	-999	-999
**633**	**A**	0.1812	-999	-999	-999
**634**	**A**	0.5766	-999	-999	-999
**635**	**G**	0.7002	-999	-999	-999
**636**	**C**	0.0062	-999	-999	-999
**637**		-999	-999	-999	-999
**638**		-999	-999	-999	-999
**639**		-999	-999	-999	-999
**640**		-999	-999	-999	-999
**641**		-999	-999	-999	-999
**642**		-999	-999	-999	-999
**643**		-999	-999	-999	-999
**644**		-999	-999	-999	-999
**645**		-999	-999	-999	-999
**646**		-999	-999	-999	-999
**647**		-999	-999	-999	-999
**648**		-999	-999	-999	-999
**649**		-999	-999	-999	-999

**650**	-999	-999	-999	-999
**651**	-999	-999	-999	-999
**652**	-999	-999	-999	-999
**653**	-999	-999	-999	-999
**654**	-999	-999	-999	-999
**655**	-999	-999	-999	-999
**656**	-999	-999	-999	-999
**657**	-999	-999	-999	-999
**658**	-999	-999	-999	-999
**659**	-999	-999	-999	-999
**660**	-999	-999	-999	-999
**661**	-999	-999	-999	-999
**662**	-999	-999	-999	-999
**663**	-999	-999	-999	-999
**664**	-999	-999	-999	-999
**665**	-999	-999	-999	-999
**666**	-999	-999	-999	-999
**667**	-999	-999	-999	-999
**668**	-999	-999	-999	-999
**669**	-999	-999	-999	-999
**670**	-999	-999	-999	-999
**671**	-999	-999	-999	-999
**672**	-999	-999	-999	-999
**673**	-999	-999	-999	-999
**674**	-999	-999	-999	-999
**675**	-999	-999	-999	-999
**676**	-999	-999	-999	-999
**677**	-999	-999	-999	-999
**678**	-999	-999	-999	-999
**679**	-999	-999	-999	-999
**680**	-999	-999	-999	-999
**681**	-999	-999	-999	-999
**682**	-999	-999	-999	-999
**683**	-999	-999	-999	-999
**684**	-999	-999	-999	-999
**685**	-999	-999	-999	-999
**686**	-999	-999	-999	-999
**687**	-999	-999	-999	-999
**688**	-999	-999	-999	-999
**689**	-999	-999	-999	-999
**690**	-999	-999	-999	-999

Dimer input data for RNAstructure

seqnum	seq	WT D	WT+gag D	S1+gag D	U5s+gag D	LU5AUG+gag D
7	U	-999	-999	-999	-999	-999
8	C	-999	-999	-999	-999	-999
9	U	-999	-999	-999	-999	-999
10	G	-999	-999	-999	-999	-999
11	G	-999	-999	-999	-999	-999
12	U	-999	-999	-999	-999	-999
13	U	-999	-999	-999	-999	-999
14	A	-999	-999	-999	-999	-999
15	G	-999	-999	-999	-999	-999
16	A	-999	-999	-999	-999	-999
17	C	-999	-999	-999	-999	-999
18	C	-999	-999	-999	-999	-999
19	A	-999	-999	-999	-999	-999
20	G	-999	-999	-999	-999	-999
21	A	-999	0.4629	0.3059	-999	-999
22	U	0.9579	0.2719	-0.0052	-999	-999
23	C	0.8301	0.403	0.3346	-999	2.3505
24	U	0.4457	0.2255	-0.0076	-999	1.2445
25	G	0.0851	0.0782	0.2188	-999	0.9345
26	A	0.0933	0.0878	0.3235	-999	-0.018
27	G	-0.0438	0.5136	0.0087	-999	0.6079
28	C	0.2676	0.2291	0.0542	-999	0.1765
29	C	0.1665	0.4099	0.0523	-999	0.5832
30	U	0.9092	0.1655	0.4259	-999	0.0399
31	G	0.9601	0.8983	1.1875	-999	1.5687
32	G	0.3506	0.754	0.0095	-999	0.4559
33	G	0.5436	0.8336	1.4459	-999	0.1727
34	A	0.5929	0.8276	0.8359	-999	-0.132
35	G	0.2198	0.1303	0.4028	-999	2.0345
36	C	0.0561	0.0229	0.1224	-999	0.1156
37	U	0.1152	-0.017	0.1907	-999	0.1862
38	C	0.0861	0.0073	0.0712	-999	0.289
39	U	0.2023	0.0292	0.0571	-999	0.0185
40	C	0.0532	0.0506	0.0053	-999	0.2846
41	U	0.9397	0.1264	0.5961	-999	0.0227
42	G	0.1597	0.0977	0.2984	-999	0.1259
43	G	0.1143	0.0397	0.2635	-999	0.4799
44	C	0.0318	0.0361	-0.0369	-999	0.4153
45	U	0.0619	0.1964	0.3341	0.6411	0.3292
46	A	0.1568	0.0852	0.3067	0.2638	0.2554
47	A	0.0654	-0.051	0.0692	0.4051	0.256

**48**	**C**	0.0243	-0.008	0.0174	0.477	0.3033
**49**	**U**	0.1516	0.0205	0.1604	0.2971	0.1841
**50**	**A**	0.2318	-0.115	0.1889	0.2472	-3E-04
**51**	**G**	-0.1164	0.0808	0.3482	0.3595	0.074
**52**	**G**	0.2325	-0.052	0.2628	0.172	0.3932
**53**	**G**	1.6893	0.1394	0.3569	-0.018	0.3055
**54**	**A**	2.2913	0.0156	0.2302	0.3168	0.3304
**55**	**A**	-3.8137	0.7198	0.9009	0.4924	-0.042
**56**	**C**	0.1431	0.0162	0.2054	0.7302	1.1702
**57**	**C**	2.456	-0.011	0.77	0.3083	0.4694
**58**	**C**	-2.1164	-0.002	0.8193	-0.555	0.8613
**59**	**A**	-2.9492	0.0859	0.0134	0.4311	1.1397
**60**	**C**	-0.038	0.86	0.4346	0.6978	0.05
**61**	**U**	2.9625	0.8854	1.026	-0.321	0.5307
**62**	**G**	0.2604	0.2393	0.4208	0.2059	0.4031
**63**	**C**	-0.0753	0.0045	-0.093	0.0529	0.0511
**64**	**U**	0.0539	0.0384	-0.0229	-0.072	0.1597
**65**	**U**	0.2467	4.272	0.656	-0.927	0.3266
**66**	**A**	-0.0095	0.4542	0.0982	0.233	2.4529
**67**	**A**	0.4644	0.3367	0.5131	0.3426	0.0857
**68**	**G**	0.0695	0.0026	0.1064	-0.181	0.4362
**69**	**C**	0.1436	0.0656	0.1128	0.8336	0.4881
**70**	**C**	0.0267	0.0026	0.0221	-0.598	0.1653
**71**	**U**	0.2061	0.1008	0.1638	0.0919	0.2822
**72**	**C**	0.2847	-0.016	0.5996	-0.219	0.5023
**73**	**A**	0.4377	0.3688	0.9194	-0.304	0.9068
**74**	**A**	0.2591	1.3037	0.7318	-0.099	0.3286
**75**	**U**	0.7332	1.4076	0.0871	0.1456	0.244
**76**	**A**	0.4302	0.1037	0.1377	0.2061	1.9424
**77**	**A**	0.4628	0.4967	0.3851	0.6558	0.1387
**78**	**A**	0.2256	0.2089	0.277	0.6642	0.1116
**79**	**G**	0.0895	0.0564	0.0128	0.1104	0.529
**80**	**C**	0.1501	-0.033	0.0534	0.0544	0.2565
**81**	**U**	0.6589	0.0907	0.1623	-0.055	0.2384
**82**	**U**	6.75	0.2276	1.6138	0.2943	-0.405
**83**	**G**	0.6424	1.0402	0.3697	0.4978	-2.415
**84**	**C**	0.5932	0.2313	0.1527	0.2188	-0.077
**85**	**C**	0.2677	0.0617	0.1599	-0.248	0.256
**86**	**U**	0.0689	-0.044	0.0054	0.1138	0.1946
**87**	**U**	0.356	0.1001	0.6278	0.2	0.2218
**88**	**G**	0.0544	0.3592	0.2519	-0.026	0.2077
**89**	**A**	0.071	0.5165	0.1947	0.3486	-0.015
**90**	**G**	0.2034	0.2218	0.0507	0.3022	0.5729

**91**	**U**	0.6096	0.3848	0.4889	0.1405	0.1716
**92**	**G**	0.2409	0.1025	0.0481	0.0518	0.5884
**93**	**C**	0.2412	0.0961	0.07	0.0053	0.218
**94**	**U**	0.1415	0.0617	0.1629	-0.061	0.1558
**95**	**U**	0.0709	0.0797	-0.0978	-0.125	0.1713
**96**	**C**	0.5557	0.932	0.9678	-0.721	0.3115
**97**	**A**	0.1738	0.1197	0.8197	0.1954	3.3199
**98**	**A**	0.0512	-0.177	0.0671	-1.639	0.1553
**99**	**G**	0.3415	2.1851	-0.1669	-0.159	0.1183
**100**	**U**	0.3064	4.7665	0.4645	-0.719	0.5084
**101**	**A**	0.1273	1.6993	0.1018	0.1698	12.786
**102**	**G**	-0.0705	1.0107	1.2849	0.1975	-1.009
**103**	**U**	0.5762	0.5454	0.4093	-0.277	0.9418
**104**	**G**	0.0054	-0.015	1.4229	0.1106	2.044
**105**	**U**	1.6269	0.5289	-0.0567	0.1367	1.1418
**106**	**G**	0.5451	0.612	0.3795	-0.162	0.3013
**107**	**U**	0.4961	0.5579	0.3182	0.2456	0.1928
**108**	**G**	0.2322	0.0614	0.1892	0.0362	0.3023
**109**	**C**	0.9559	0.1361	0.1623	0.1914	-0.03
**110**	**C**	0.0289	0.0115	0.0815	-0.015	-0.717
**111**	**C**	0.4232	0.196	0.6235	0.0342	-0.027
**112**	**G**	0.1278	0.1177	0.183	-0.125	-0.135
**113**	**U**	0.7371	0.2459	0.3965	0.3004	0.0009
**114**	**C**	0.0172	0.1172	0.1491	0.0198	-0.52
**115**	**U**	0.1889	0.1346	1.1488	0.7835	0.0833
**116**	**G**	0.3545	0.1704	-0.2701	-0.927	0.5195
**117**	**U**	0.3265	0.1697	-3.6398	-1.586	0.5504
**118**	**U**	0.8444	0.6195	6.2958	-6.307	0.272
**119**	**G**	0.3213	0.0025	19.547	5.5497	0.6755
**120**	**U**	1.339	0.7396	19.947	6.9795	0.2007
**121**	**G**	0.0929	-0.131	2.4294	0.2834	0.0106
**122**	**U**	0.7411	0.6348	2.2369	-0.717	-0.002
**123**	**G**	0.1779	0.2	0.3248	0.4308	0.2335
**124**	**A**	0.2877	0.0923	0.3787	0.1018	0.0105
**125**	**C**	0.1201	0.0169	-0.1128	0.0396	0.317
**126**	**U**	0.0481	-0.068	0.0026	0.0243	0.1606
**127**	**C**	0.3524	0.0631	0.1587	-0.092	0.1153
**128**	**U**	0.3221	0.241	0.1777	0.4916	-0.086
**129**	**G**	0.3585	0.3191	0.5962	0.0507	0.2376
**130**	**G**	0.1109	-0.035	0.2326	0.8953	0.3605
**131**	**U**	0.7539	1.6067	1.3066	-1.136	0.8177
**132**	**A**	0.3817	0.905	0.936	0.3634	0.2478
**133**	**A**	0.2369	0.0901	0.107	0.1277	0.2837

**134**	**C**	0.0772	0.1692	0.2717	-0.225	0.3045
**135**	**U**	0.1332	0.3981	0.5783	-0.183	0.5434
**136**	**A**	0.2013	0.2547	0.6743	0.1911	0.5018
**137**	**G**	0.3071	0.3488	0.2302	-0.027	0.3301
**138**	**A**	0.0965	0.0027	0.1995	0.284	0.0687
**139**	**G**	0.1895	0.2256	0.1178	-0.055	0.4361
**140**	**A**	0.2781	-0.065	0.2342	0.0065	-0.232
**141**	**U**	6.9221	0.1506	0.3723	0.5096	-4.031
**142**	**C**	0.6065	0.2775	0.219	-0.047	-4.956
**143**	**C**	0.3391	0.1346	0.149	0.0613	-0.183
**144**	**C**	0.0076	-0.018	0.0361	0.2327	0.2177
**145**	**U**	0.1313	0.2072	0.5371	-0.075	0.1501
**146**	**C**	0.0319	0.0104	0.781	-0.226	0.3912
**147**	**A**	-0.0055	0.7343	0.3842	-0.318	0.4901
**148**	**G**	0.1941	0.6706	-0.0042	0.2785	0.3997
**149**	**A**	0.742	3.4939	0.5405	-1.058	-0.13
**150**	**C**	3.4733	1.7059	-0.0805	5.0901	-1.291
**151**	**C**	5.1959	0.5562	0.3028	0.8743	3.3673
**152**	**C**	8.1092	0.7652	0.4126	-0.129	2.2012
**153**	**U**	1.3249	0.2154	0.3712	0.6106	-0.652
**154**	**U**	0.2094	0.0542	0.1994	0.1561	0.6812
**155**	**U**	2.0195	0.9682	1.1289	0.6602	-0.051
**156**	**U**	1.752	2.9993	0.8521	1.4362	-1.661
**157**	**A**	0.4324	0.8402	0.8457	1.0239	2.3317
**158**	**G**	0.1072	0.0703	0.0958	-0.022	0.4881
**159**	**U**	0.1277	0.2608	0.2617	0.2462	0.0851
**160**	**C**	0.4005	0.4411	0.8547	0.3296	0.3044
**161**	**A**	0.3182	0.4436	0.5042	0.9084	0.7118
**162**	**G**	0.2285	0.4428	0.1378	0.1219	0.134
**163**	**U**	0.1675	0.0053	0.1066	0.2186	0.2313
**164**	**G**	0.8952	0.534	0.4948	0.0481	0.2609
**165**	**U**	1.0341	0.9568	0.9022	0.5301	-1.367
**166**	**G**	0.7587	0.555	0.505	0.079	-1.147
**167**	**G**	0.1584	0.3744	0.47	-0.713	0.0946
**168**	**A**	0.5484	0.6769	0.5139	0.7602	0.1884
**169**	**A**	0.3356	-0.101	0.4773	0.4567	0.5494
**170**	**A**	0.5359	0.9555	0.5718	1.1738	0.146
**171**	**A**	0.0628	0.2111	0.0722	0.2496	0.6233
**172**	**U**	0.1961	0.1189	0.2136	-0.009	0.3201
**173**	**C**	0.0463	-0.01	0.0122	0.3253	0.0811
**174**	**U**	0.1468	0.0273	0.1111	0.1299	0.0676
**175**	**C**	0.0352	0.0215	0.1673	0.2858	0.1106
**176**	**U**	0.3386	1.3432	1.0388	0.1645	0.0583

**177**	**A**	0.2576	0.6114	0.5354	0.2857	0.2802
**178**	**G**	0.4656	0.3285	0.6288	-0.091	0.1041
**179**	**C**	0.7752	0.4297	1.921	-0.919	0.4279
**180**	**A**	0.423	0.9721	1.0513	0.4914	1.6732
**181**	**G**	-0.0591	0.2395	0.1028	-0.2	0.434
**182**	**U**	0.407	0.4511	0.6726	0.4061	0.5143
**183**	**G**	0.2534	-0.094	0.9818	0.0633	1.0929
**184**	**G**	-0.0181	0.129	0.7074	1.6735	0.5048
**185**	**C**	0.3717	0.2514	0.0843	0.1299	1.1985
**186**	**G**	0.147	0.2851	0.3584	0.4147	0.3615
**187**	**C**	0.1677	0.0004	0.1077	0.1662	-0.07
**188**	**C**	0.087	0.1032	0.112	0.3458	-0.036
**189**	**C**	-0.043	0.2389	0.2515	0.1256	0.121
**190**	**G**	0.0863	0.6041	0.5067	-0.168	0.2168
**191**	**A**	0.325	1.1505	0.7017	0.551	0.1909
**192**	**A**	0.3077	1.4054	0.0885	0.2336	0.4444
**193**	**C**	1.0466	0.8069	0.2847	-0.229	0.814
**194**	**A**	0.525	0.3322	0.3845	0.9159	1.5925
**195**	**G**	-0.2277	0.0084	0.0298	0.2891	0.0983
**196**	**G**	-3.3617	-0.071	0.3817	0.7458	0.1159
**197**	**G**	3.2302	0.1927	0.248	-0.095	0.9074
**198**	**A**	1.9714	0.1827	0.1911	0.2112	-1.399
**199**	**C**	0.2314	-0.034	0.2301	1.4736	-0.131
**200**	**C**	0.1052	0.2244	0.2119	1.8821	0.3968
**201**	**U**	0.6412	0.8744	1.4363	0.8229	1.7611
**202**	**G**	0.4881	0.423	0.8053	0.047	2.6339
**203**	**A**	0.1825	0.1071	0.3529	0.2518	0.629
**204**	**A**	0.164	0.1879	0.4235	0.6444	0.0728
**205**	**A**	0.2884	0.6739	0.5825	0.1523	-0.139
**206**	**G**	0.2706	0.4084	0.347	-0.497	-0.329
**207**	**C**	0.5426	2.5527	0.5385	-0.342	0.614
**208**	**G**	0.2026	0.1492	0.1862	0.4238	1.5882
**209**	**A**	0.2842	0.2735	0.2209	0.2082	0.2087
**210**	**A**	0.2339	0.3517	0.2337	-0.005	0.4988
**211**	**A**	0.5666	0.6177	0.2311	1.7169	0.1748
**212**	**G**	0.5482	0.7499	0.1529	1.0026	0.2497
**213**	**G**	-0.1526	0.2004	0.2895	-0.391	1.0295
**214**	**G**	0.2888	0.3841	0.591	0.4437	0.1058
**215**	**A**	0.2828	0.5562	0.4034	0.2497	0.5939
**216**	**A**	0.2853	0.5428	0.5685	0.1852	0.2745
**217**	**A**	0.0487	0.0966	0.1851	0.1068	0.3509
**218**	**C**	0.1396	0.007	0.2504	-0.036	0.3212
**219**	**C**	0.2495	0.5936	1.4096	-0.589	0.4255

**220**	**A**	0.5099	0.3562	0.4773	0.3131	0.7888
**221**	**G**	0.1521	0.0742	0.217	0.047	0.5465
**222**	**A**	0.3926	0.0331	0.321	0.3816	0.1581
**223**	**G**	-0.1988	-0.215	0.1145	0.1161	0.5335
**224**	**G**	0.7656	0.6207	0.8424	0.5823	0.1166
**225**	**A**	0.0326	0.1584	0.2736	0.3282	0.0356
**226**	**G**	0.5731	0.4722	0.5682	0.4148	0.4665
**227**	**C**	-0.0296	-0.003	0.0208	0.0077	-0.042
**228**	**U**	0.174	0.112	0.2824	0.2784	0.0273
**229**	**C**	-0.0286	0.0344	0.0498	0.0903	-0.272
**230**	**U**	0.2814	0.5199	0.24	0.1343	-0.018
**231**	**C**	0.3446	0.4847	0.3904	0.1451	0.259
**232**	**U**	0.0454	0.0107	0.0057	-0.031	0.1485
**233**	**C**	0.1364	0.3437	0.2165	0.121	0.1339
**234**	**G**	0.1284	0.1049	0.0593	-0.52	0.3225
**235**	**A**	0.161	0.1872	0.5007	-0.248	-0.072
**236**	**C**	0.2696	0.2724	0.2992	-0.062	0.2139
**237**	**G**	0.3039	0.0257	0.1094	0.2795	0.7969
**238**	**C**	1.977	1.0331	1.6799	-0.436	0.5378
**239**	**A**	0.0588	0.5373	0.3465	-0.001	4.3698
**240**	**G**	1.5844	3.161	2.8726	0.8464	0.6296
**241**	**G**	0.3374	1.476	0.7368	3.234	1.4955
**242**	**A**	0.4554	0.5423	0.6072	0.4892	0.4481
**243**	**C**	0.1486	0.1649	0.2069	0.4334	0.6173
**244**	**U**	0.149	-0.015	0.0572	-0.235	0.3019
**245**	**C**	0.7086	0.4373	0.5182	0.0899	-0.061
**246**	**G**	-0.2523	0.0055	0.3768	0.391	-1.929
**247**	**G**	0.584	0.2237	0.3298	0.3048	-0.373
**248**	**C**	0.0052	0.0033	0.0658	-0.285	0.6699
**249**	**U**	0.1584	0.0922	0.1768	-0.104	0.0435
**250**	**U**	0.1427	0.1628	0.0755	0.0897	-0.006
**251**	**G**	0.15	-0.019	0.1534	0.2445	-0.098
**252**	**C**	0.1411	0.0775	-0.0163	-0.052	0.0435
**253**	**U**	0.2919	0.2187	0.1675	0.1255	0.273
**254**	**G**	0.1133	0.2564	-0.096	0.096	0.4372
**255**	**A**	0.3847	0.8285	0.6167	0.5256	0.1907
**256**	**A**	0.6729	0.9811	1.1971	1.7204	0.2328
**257**	**G**	-999	-999	-999	-999	-999
**258**	**C**	-999	-999	-999	-999	-999
**259**	**G**	-999	-999	-999	-999	-999
**260**	**C**	-999	-999	-999	-999	-999
**261**	**G**	-999	-999	-999	-999	-999
**262**	**C**	-999	-999	-999	-999	-999

**263**	**A**	0.0712	0.018	0.0344	-0.081	0.1657
**264**	**C**	0.2459	0.1657	0.3535	0.2252	0.3805
**265**	**G**	-0.0198	-0.041	0.0881	0.3058	0.2662
**266**	**G**	-0.0166	0.1348	0.1701	0.2146	0.6024
**267**	**C**	0.0002	0.2972	0.3666	0.055	0.0883
**268**	**A**	0.2106	-0.01	0.1256	-0.138	1.119
**269**	**A**	0.1874	0.0584	0.1811	0.4027	0.0636
**270**	**G**	0.0835	0.1954	0.1945	-0.066	0.3295
**271**	**A**	0.2885	0.3773	0.636	0.467	0.776
**272**	**G**	0.5634	1.3923	2.0563	1.1425	0.1577
**273**	**G**	0.9332	2.1994	0.5046	2.5654	1.9112
**274**	**C**	0.6394	0.5319	0.2918	0.253	0.6197
**275**	**G**	0.4373	0.0898	0.0297	0.1846	0.9615
**276**	**A**	0.5026	0.0667	0.1917	0.2488	0.7826
**277**	**G**	0.1575	0.097	0.1163	0.1112	0.1616
**278**	**G**	0.2732	0.1032	0.3117	0.4064	0.246
**279**	**G**	0.2827	0.4252	0.4761	0.3779	-0.024
**280**	**G**	0.3776	0.243	0.0936	0.3207	0.9095
**281**	**C**	0.2705	0.0308	0.0471	0.3399	0.5552
**282**	**G**	0.4022	0.5538	0.5237	0.149	-0.02
**283**	**G**	0.0537	0.1774	0.0245	0.3304	0.6385
**284**	**C**	0.0769	0.6984	0.4396	0.1316	0.1248
**285**	**G**	0.2826	0.2475	0.1513	-0.124	0.559
**286**	**A**	0.2109	0.0472	0.0602	0.134	0.1557
**287**	**C**	0.5152	0.1923	0.2406	0.4752	0.2896
**288**	**U**	0.8745	0.6114	0.8077	0.6325	0.5818
**289**	**G**	0.2131	0.4815	0.2222	-0.265	0.5872
**290**	**G**	0.224	0.7369	0.4824	0.9051	0.2286
**291**	**U**	0.6607	0.3515	-0.0921	-0.009	0.6194
**292**	**G**	0.3321	0.9236	0.8392	0.7386	-0.193
**293**	**A**	0.0748	0.0668	0.048	-0.022	0.5654
**294**	**G**	-0.44	-0.033	0.2573	0.1861	0.0875
**295**	**U**	0.7019	1.1113	0.633	-0.473	0.4088
**296**	**A**	0.3476	0.4587	0.4982	-0.159	2.0785
**297**	**C**	0.6051	-0.237	0.1796	-0.004	0.3761
**298**	**G**	1.505	0.2194	0.2922	-0.219	0.0735
**299**	**C**	0.9866	0.1461	0.1016	-0.594	-0.755
**300**	**C**	1.8224	0.2644	0.4982	-0.674	-0.148
**301**	**A**	0.8196	0.3888	0.8561	0.1192	-0.202
**302**	**A**	0.4694	0.0979	0.2457	0.1421	-0.749
**303**	**A**	-0.2743	0.1695	0.3556	0.511	-0.225
**304**	**A**	0.1638	0.5588	0.5929	0.4683	-0.264
**305**	**A**	0.1294	0.6067	0.7624	0.974	-0.744

**306**	**U**	-0.6089	0.0765	0.2852	0.5051	0.3603
**307**	**U**	-1.3094	0.1347	0.1921	0.0856	0.868
**308**	**U**	-0.94	0.1301	0.0918	0.0996	0.6881
**309**	**U**	1.0105	1.852	-0.4502	0.4654	0.2692
**310**	**G**	0.2768	0.9382	0.0092	0.3109	1.1655
**311**	**A**	0.3268	0.1879	0.495	0.0803	0.1949
**312**	**C**	-0.1623	-0.035	0.1799	-0.007	0.3263
**313**	**U**	0.4727	0.3921	0.0945	-0.29	0.1594
**314**	**A**	0.247	0.0816	0.4065	0.2258	0.533
**315**	**G**	0.1901	0.0735	0.0787	0.1306	0.0141
**316**	**C**	0.0462	-0.074	0.236	0.0906	0.0844
**317**	**G**	0.3457	0.0582	0.3252	0.8387	0.4991
**318**	**G**	1.1977	0.3514	0.563	1.0288	-0.029
**319**	**A**	1.0772	0.8849	0.7397	1.381	0.4872
**320**	**G**	-0.2721	0.7829	0.9878	1.0483	-1.3
**321**	**G**	0.334	0.1466	0.1671	-0.112	1.7729
**322**	**C**	0.5327	0.0686	0.051	1.8234	0.0745
**323**	**U**	-1.334	0.2978	0.2695	0.0449	0.1374
**324**	**A**	0.599	-0.03	0.4887	0.2155	0.2896
**325**	**G**	0.2241	-0.013	0.0605	0.2483	0.2339
**326**	**A**	0.1729	0.161	0.3564	0.2472	0.0527
**327**	**A**	-0.084	0.0398	0.2661	0.0728	0.2016
**328**	**G**	0.6675	-0.016	0.292	0.2169	0.0541
**329**	**G**	0.2597	0.3342	0.1705	0.25	0.6239
**330**	**A**	0.4616	-0.002	0.18	0.523	0.1145
**331**	**G**	0.2984	-0.135	0.3539	0.1271	0.038
**332**	**A**	0.6152	0.2421	0.8721	1.3652	0.1842
**333**	**G**	0.3437	0.2472	0.4841	0.1343	0.2503
**334**	**A**	0.411	0.0971	0.3915	0.0181	0.1101
**335**	**G**	0.2288	0.1327	0.1954	0.101	0.8214
**336**	**A**	0.0082	0.153	0.0977	-0.231	0.02
**337**	**U**	0.4665	0.0493	0.2103	0.4092	0.1226
**338**	**G**	0.3527	0.0631	0.4649	0.4755	0.1023
**339**	**G**	0.1655	-0.067	0.2394	0.3665	0.9619
**340**	**G**	0.2967	0.134	0.0321	0.1501	-0.078
**341**	**U**	0.4318	0.2038	0.6964	0.2985	0.4575
**342**	**G**	-0.0206	0.0965	0.3164	0.0161	0.0902
**343**	**C**	-0.1186	0.064	0.1512	0.2129	0.019
**344**	**G**	0.2673	0.1309	0.2921	0.4093	-1.079
**345**	**A**	0.282	-0.006	0.7852	0.8513	-3.687
**346**	**G**	0.3795	0.0588	0.5113	0.8518	-2.021
**347**	**A**	3.2102	0.1234	0.8258	-1.37	0.0825
**348**	**G**	-0.0686	0.1872	0.8741	1.0503	-1.65

**349**	**C**	0.6741	-0.057	0.1916	-0.763	0.3542
**350**	**G**	0.1203	0.0821	0.9707	-1.168	0.2508
**351**	**U**	-4.054	-0.047	0.0255	1.1176	0.2453
**352**	**C**	0.3318	-0.037	0.9612	0.1519	0.2377
**353**	**A**	1.2242	-0.032	4.2996	-2.777	0.8667
**354**	**G**	0.8854	0.2262	0.1089	0.9989	0.3583
**355**	**U**	0.582	2.2708	0.0969	-0.941	0.1284
**356**	**A**		0.97	6.3146	-0.39	-999
**357**	**U**		-0.055	0.1854	0.6488	-999
**358**	**U**		2.1204	0.8853	0.0556	-999
**359**	**A**		0.4569	2.7137	0.288	-999
**360**	**A**		0.359	0.6272	0.9534	-999
**361**	**G**		0.3562	0.0025	-0.368	-999
**362**	**C**		0.2017	-999	0.6616	-999
**363**	**G**		-0.129	-999	-999	-999
**364**	**G**		-0.014	-999	-999	-999
**365**	**G**		0.3576	-999	-999	-999
**366**	**G**		-0.259	-999	-999	-999
**367**	**G**		0.987	-999	-999	-999
**368**	**A**		-0.049	-999	-999	-999
**369**	**G**		0.0842	-999	-999	-999
**370**	**A**		0.0955	-999	-999	-999
**371**	**A**		0.0954	-999	-999	-999
**372**	**U**		0.479	-999	-999	-999
**373**	**U**		1.124	-999	-999	-999
**374**	**A**		0.0941	-999	-999	-999
**375**	**G**		0.2381	-999	-999	-999
**376**	**A**		-0.013	-999	-999	-999
**377**	**U**		0.0687	-999	-999	-999
**378**	**C**		-0.095	-999	-999	-999
**379**	**G**		0.7041	-999	-999	-999
**380**	**C**		0.1904	-999	-999	-999
**381**	**G**		-0.018	-999	-999	-999
**382**	**A**		0.0198	-999	-999	-999
**383**	**U**		0.511	-999	-999	-999
**384**	**G**		-0.222	-999	-999	-999
**385**	**G**		1.9666	-999	-999	-999
**386**	**G**		-0.211	-999	-999	-999
**387**	**A**		0.2122	-999	-999	-999
**388**	**A**		0.0357	-999	-999	-999
**389**	**A**		0.0456	-999	-999	-999
**390**	**A**		0.3164	-999	-999	-999
**391**	**A**		0.0646	-999	-999	-999

**392**	**A**	0.0964	-999	-999	-999
**393**	**U**	0.0044	-999	-999	-999
**394**	**U**	0.2085	-999	-999	-999
**395**	**C**	0.45	-999	-999	-999
**396**	**G**	0.201	-999	-999	-999
**397**	**G**	0.3302	-999	-999	-999
**398**	**U**	0.3584	-999	-999	-999
**399**	**U**	0.4637	-999	-999	-999
**400**	**A**	0.4495	-999	-999	-999
**401**	**A**	0.2208	-999	-999	-999
**402**	**G**	0.3603	-999	-999	-999
**403**	**G**	0.0587	-999	-999	-999
**404**	**C**	0.0541	-999	-999	-999
**405**	**C**	0.17	-999	-999	-999
**406**	**A**	0.1669	-999	-999	-999
**407**	**G**	0.1536	-999	-999	-999
**408**	**G**	0.2497	-999	-999	-999
**409**	**G**	-1.285	-999	-999	-999
**410**	**G**	1.1345	-999	-999	-999
**411**	**G**	0.7721	-999	-999	-999
**412**	**A**	0.2995	-999	-999	-999
**413**	**A**	0.2727	-999	-999	-999
**414**	**A**	0.654	-999	-999	-999
**415**	**G**	0.1704	-999	-999	-999
**416**	**A**	0.2186	-999	-999	-999
**417**	**A**	0.2609	-999	-999	-999
**418**	**A**	0.1065	-999	-999	-999
**419**	**A**	0.0708	-999	-999	-999
**420**	**A**	0.3029	-999	-999	-999
**421**	**A**	0.6373	-999	-999	-999
**422**	**U**	0.6746	-999	-999	-999
**423**	**A**	0.4165	-999	-999	-999
**424**	**U**	0.8152	-999	-999	-999
**425**	**A**	0.5115	-999	-999	-999
**426**	**A**	0.2275	-999	-999	-999
**427**	**A**	0.4735	-999	-999	-999
**428**	**U**	0.2888	-999	-999	-999
**429**	**U**	1.4295	-999	-999	-999
**430**	**A**	-0.167	-999	-999	-999
**431**	**A**	0.0327	-999	-999	-999
**432**	**A**	0.0796	-999	-999	-999
**433**	**A**	0.2155	-999	-999	-999
**434**	**C**	0.5603	-999	-999	-999

**435**	**A**	0.3531	-999	-999	-999
**436**	**U**	0.2479	-999	-999	-999
**437**	**A**	0.5307	-999	-999	-999
**438**	**U**	0.741	-999	-999	-999
**439**	**A**	0.5346	-999	-999	-999
**440**	**G**	0.8913	-999	-999	-999
**441**	**U**	0.6115	-999	-999	-999
**442**	**A**	-0.336	-999	-999	-999
**443**	**U**	0.7901	-999	-999	-999
**444**	**G**	-0.09	-999	-999	-999
**445**	**G**	0.0798	-999	-999	-999
**446**	**G**	0.0848	-999	-999	-999
**447**	**C**	-0.123	-999	-999	-999
**448**	**A**	-0.008	-999	-999	-999
**449**	**A**	0.1316	-999	-999	-999
**450**	**G**	0.1877	-999	-999	-999
**451**	**C**	0.2601	-999	-999	-999
**452**	**A**	-0.005	-999	-999	-999
**453**	**G**	0.9273	-999	-999	-999
**454**	**G**	1.4877	-999	-999	-999
**455**	**G**	1.063	-999	-999	-999
**456**	**A**	0.5303	-999	-999	-999
**457**	**G**	0.0901	-999	-999	-999
**458**	**C**	0.2106	-999	-999	-999
**459**	**U**	0.488	-999	-999	-999
**460**	**A**	0.0825	-999	-999	-999
**461**	**G**	0.3725	-999	-999	-999
**462**	**A**	0.0153	-999	-999	-999
**463**	**A**	0.245	-999	-999	-999
**464**	**C**	0.1564	-999	-999	-999
**465**	**G**	0.0608	-999	-999	-999
**466**	**A**	0.1132	-999	-999	-999
**467**	**U**	0.1327	-999	-999	-999
**468**	**U**	0.1181	-999	-999	-999
**469**	**C**	0.0681	-999	-999	-999
**470**	**G**	0.0325	-999	-999	-999
**471**	**C**	0.0541	-999	-999	-999
**472**	**A**	0.2745	-999	-999	-999
**473**	**G**	0.0956	-999	-999	-999
**474**	**U**	0.1359	-999	-999	-999
**475**	**U**	0.0792	-999	-999	-999
**476**	**A**	0.2408	-999	-999	-999
**477**	**A**	0.3328	-999	-999	-999

**478**	**U**	-0.082	-999	-999	-999
**479**	**C**	0.1032	-999	-999	-999
**480**	**C**	-0.012	-999	-999	-999
**481**	**U**	0.1087	-999	-999	-999
**482**	**G**	-0.003	-999	-999	-999
**483**	**G**	0.2389	-999	-999	-999
**484**	**C**	-0.299	-999	-999	-999
**485**	**C**	2.6168	-999	-999	-999
**486**	**U**	1.6518	-999	-999	-999
**487**	**G**	0.7538	-999	-999	-999
**488**	**U**	0.6793	-999	-999	-999
**489**	**U**	1.8009	-999	-999	-999
**490**	**A**	2.1793	-999	-999	-999
**491**	**G**	-0.014	-999	-999	-999
**492**	**A**	0.0785	-999	-999	-999
**493**	**A**	0.0675	-999	-999	-999
**494**	**A**	0.1872	-999	-999	-999
**495**	**C**	1.1865	-999	-999	-999
**496**	**A**	0.3723	-999	-999	-999
**497**	**U**	0.3854	-999	-999	-999
**498**	**C**	2.2257	-999	-999	-999
**499**	**A**	0.2757	-999	-999	-999
**500**	**G**	0.433	-999	-999	-999
**501**	**A**	0.0759	-999	-999	-999
**502**	**A**	-0.117	-999	-999	-999
**503**	**G**	0.4407	-999	-999	-999
**504**	**G**	-0.09	-999	-999	-999
**505**	**C**	-0.185	-999	-999	-999
**506**	**U**	0.0544	-999	-999	-999
**507**	**G**	0.2743	-999	-999	-999
**508**	**U**	0.5101	-999	-999	-999
**509**	**A**	1.0786	-999	-999	-999
**510**	**G**	1.0457	-999	-999	-999
**511**	**A**	0.7644	-999	-999	-999
**512**	**C**	0.5495	-999	-999	-999
**513**	**A**	0.3037	-999	-999	-999
**514**	**A**	0.0402	-999	-999	-999
**515**	**A**	0.2133	-999	-999	-999
**516**	**U**	0.5252	-999	-999	-999
**517**	**A**	-0.066	-999	-999	-999
**518**	**C**	-0.019	-999	-999	-999
**519**	**U**	0.1828	-999	-999	-999
**520**	**G**	0.1646	-999	-999	-999

**521**	**G**	0.5436	-999	-999	-999
**522**	**G**	0.548	-999	-999	-999
**523**	**A**	0.3369	-999	-999	-999
**524**	**C**	0.5439	-999	-999	-999
**525**	**A**	0.4134	-999	-999	-999
**526**	**G**	-0.011	-999	-999	-999
**527**	**C**	-0.043	-999	-999	-999
**528**	**U**	0.1905	-999	-999	-999
**529**	**A**	0.4294	-999	-999	-999
**530**	**C**	0.6731	-999	-999	-999
**531**	**A**	0.291	-999	-999	-999
**532**	**A**	0.134	-999	-999	-999
**533**	**C**	-0.005	-999	-999	-999
**534**	**C**	1.664	-999	-999	-999
**535**	**A**	0.0163	-999	-999	-999
**536**	**U**	0.2667	-999	-999	-999
**537**	**C**	0.1651	-999	-999	-999
**538**	**C**	0.4551	-999	-999	-999
**539**	**C**	0.0312	-999	-999	-999
**540**	**U**	0.1062	-999	-999	-999
**541**	**U**	0.5493	-999	-999	-999
**542**	**C**	0.7757	-999	-999	-999
**543**	**A**	0.0857	-999	-999	-999
**544**	**G**	0.1021	-999	-999	-999
**545**	**A**	0.4225	-999	-999	-999
**546**	**C**	0.7865	-999	-999	-999
**547**	**A**	0.0769	-999	-999	-999
**548**	**G**	0.2404	-999	-999	-999
**549**	**G**	0.0157	-999	-999	-999
**550**	**A**	0.03	-999	-999	-999
**551**	**U**	0.2371	-999	-999	-999
**552**	**C**	0.8812	-999	-999	-999
**553**	**A**	0.3	-999	-999	-999
**554**	**G**	0.3825	-999	-999	-999
**555**	**A**	0.3058	-999	-999	-999
**556**	**A**	-0.044	-999	-999	-999
**557**	**G**	0.6213	-999	-999	-999
**558**	**A**	-0.099	-999	-999	-999
**559**	**A**	0.1814	-999	-999	-999
**560**	**C**	-0.077	-999	-999	-999
**561**	**U**	0.2765	-999	-999	-999
**562**	**U**	2.2894	-999	-999	-999
**563**	**A**	1.0245	-999	-999	-999

**564**	**G**	0.3622	-999	-999	-999
**565**	**A**	0.2552	-999	-999	-999
**566**	**U**	0.3005	-999	-999	-999
**567**	**C**	1.6839	-999	-999	-999
**568**	**A**	0.2299	-999	-999	-999
**569**	**U**	0.3867	-999	-999	-999
**570**	**U**	1.6162	-999	-999	-999
**571**	**A**	0.5256	-999	-999	-999
**572**	**U**	0.9038	-999	-999	-999
**573**	**A**	0.7722	-999	-999	-999
**574**	**U**	1.2698	-999	-999	-999
**575**	**A**	0.5496	-999	-999	-999
**576**	**A**	0.6755	-999	-999	-999
**577**	**U**	0.4568	-999	-999	-999
**578**	**A**	0.7628	-999	-999	-999
**579**	**C**	1.5964	-999	-999	-999
**580**	**A**	0.2315	-999	-999	-999
**581**	**G**	0.1982	-999	-999	-999
**582**	**U**	2.787	-999	-999	-999
**583**	**A**	0.2691	-999	-999	-999
**584**	**G**	0.0721	-999	-999	-999
**585**	**C**	1.0642	-999	-999	-999
**586**	**A**	0.1516	-999	-999	-999
**587**	**A**	-0.058	-999	-999	-999
**588**	**C**	-0.148	-999	-999	-999
**589**	**C**	0.0653	-999	-999	-999
**590**	**C**	-0.544	-999	-999	-999
**591**	**U**	-0.692	-999	-999	-999
**592**	**C**	-0.455	-999	-999	-999
**593**	**U**	0.6808	-999	-999	-999
**594**	**A**	-0.33	-999	-999	-999
**595**	**U**	0.0225	-999	-999	-999
**596**	**U**	0.068	-999	-999	-999
**597**	**G**	-0.031	-999	-999	-999
**598**	**U**	0.3291	-999	-999	-999
**599**	**G**	0.698	-999	-999	-999
**600**	**U**	1.8361	-999	-999	-999
**601**	**G**	-0.027	-999	-999	-999
**602**	**C**	0.2935	-999	-999	-999
**603**	**A**	0.1476	-999	-999	-999
**604**	**U**	1.066	-999	-999	-999
**605**	**C**	0.3028	-999	-999	-999
**606**	**A**	0.08	-999	-999	-999

**607**	**A**	0.0972	-999	-999	-999
**608**	**A**	0.3889	-999	-999	-999
**609**	**G**	0.7539	-999	-999	-999
**610**	**G**	0.2594	-999	-999	-999
**611**	**A**	1.4518	-999	-999	-999
**612**	**U**	0.7896	-999	-999	-999
**613**	**A**	0.1498	-999	-999	-999
**614**	**G**	0.7494	-999	-999	-999
**615**	**A**	-0.37	-999	-999	-999
**616**	**G**	-1.398	-999	-999	-999
**617**	**A**	1.7872	-999	-999	-999
**618**	**U**	0.8531	-999	-999	-999
**619**	**A**	0.0951	-999	-999	-999
**620**	**A**	0.2743	-999	-999	-999
**621**	**A**	0.1357	-999	-999	-999
**622**	**A**	0.2278	-999	-999	-999
**623**	**G**	0.3565	-999	-999	-999
**624**	**A**	-0.004	-999	-999	-999
**625**	**C**	0.5429	-999	-999	-999
**626**	**A**	0.1866	-999	-999	-999
**627**	**C**	0.3738	-999	-999	-999
**628**	**C**	0.4952	-999	-999	-999
**629**	**A**	1.1609	-999	-999	-999
**630**	**A**	-0.141	-999	-999	-999
**631**	**G**	-0.071	-999	-999	-999
**632**	**G**	0.2407	-999	-999	-999
**633**	**A**	0.0933	-999	-999	-999
**634**	**A**	1.5314	-999	-999	-999
**635**	**G**	0.2592	-999	-999	-999
**636**	**C**	0.3745	-999	-999	-999
**637**		-999	-999	-999	-999
**638**		-999	-999	-999	-999
**639**		-999	-999	-999	-999
**640**		-999	-999	-999	-999
**641**		-999	-999	-999	-999
**642**		-999	-999	-999	-999
**643**		-999	-999	-999	-999
**644**		-999	-999	-999	-999
**645**		-999	-999	-999	-999
**646**		-999	-999	-999	-999
**647**		-999	-999	-999	-999
**648**		-999	-999	-999	-999
**649**		-999	-999	-999	-999

**650**	-999	-999	-999	-999
**651**	-999	-999	-999	-999
**652**	-999	-999	-999	-999
**653**	-999	-999	-999	-999
**654**	-999	-999	-999	-999
**655**	-999	-999	-999	-999
**656**	-999	-999	-999	-999
**657**	-999	-999	-999	-999
**658**	-999	-999	-999	-999
**659**	-999	-999	-999	-999
**660**	-999	-999	-999	-999
**661**	-999	-999	-999	-999
**662**	-999	-999	-999	-999
**663**	-999	-999	-999	-999
**664**	-999	-999	-999	-999
**665**	-999	-999	-999	-999
**666**	-999	-999	-999	-999
**667**	-999	-999	-999	-999
**668**	-999	-999	-999	-999
**669**	-999	-999	-999	-999
**670**	-999	-999	-999	-999
**671**	-999	-999	-999	-999
**672**	-999	-999	-999	-999
**673**	-999	-999	-999	-999
**674**	-999	-999	-999	-999
**675**	-999	-999	-999	-999
**676**	-999	-999	-999	-999
**677**	-999	-999	-999	-999
**678**	-999	-999	-999	-999
**679**	-999	-999	-999	-999
**680**	-999	-999	-999	-999
**681**	-999	-999	-999	-999
**682**	-999	-999	-999	-999
**683**	-999	-999	-999	-999
**684**	-999	-999	-999	-999
**685**	-999	-999	-999	-999
**686**	-999	-999	-999	-999
**687**	-999	-999	-999	-999
**688**	-999	-999	-999	-999
**689**	-999	-999	-999	-999
**690**	-999	-999	-999	-999

Supplementary Table 1. Single nucleotide input SHAPE data for presented monomeric and dimeric RNA structures

A

U5 (105-115)	T	G	T	G	T	A	T	G	T	C	T
AUG (334-344)	G	C	G	T	G	G	G	T	A	G	A
Strain	G.CM.08.789_10										

U5 (105-115)	T	G	T	G	C	C	C	A	T	T	T
AUG (334-344)	G	C	G	T	G	G	G	T	A	G	A
Strain	12_BF.AR.99.ARMA159										

U5 (105-115)	T	G	T	G	C	C	C	G	T	T	T
AUG (334-344)	G	C	G	T	G	G	G	T	A	G	A
Strain	A6.BY.13.PV85										

U5 (105-115)	T	G	T	G	C	C	C	A	T	C	T
AUG (334-344)	G	C	G	T	G	G	G	T	A	G	A
Strains	A1.CM.08.886_24, A6.UA.12.DEMA112UA024, B.DO.11.DEMB11DR001, B.PH.15.DEMB15PH003, C.PK.14.DEMC14PK009, 43_02G.SA.03.J11223, 49_cpx.GM.03.N26677										

U5 (105-115)	T	C	C	A	C	T	G	G	G	C	G
AUG (334-344)	G	C	G	T	G	G	G	T	A	G	A
Strain	A1.PK.15.PK034										

U5 (105-115)	T	A	A	A	G	C	C	G	A	A	A
AUG (334-344)	G	C	G	T	G	G	G	T	A	G	A
Strain	02_AG.PK.15.PK032										

B

U5 (103 - 122)	T	G	T	G	T	G	T	A	T	G	T	-	C	T	G	T
gag (433-452)	A	C	G	A	A	C	G	G	G	T	A	T	G	A	T	-
Strain	G.CM.08.789_10															

U5 (103 - 122)	T	G	T	G	T	G	C	C	C	A	T	-	T	T	G	T
gag (433-452)	A	C	G	A	A	C	G	G	G	T	A	T	G	A	T	-
Strain	12_BF.AR.99.ARMA159															

U5 (103 - 122)	T	G	T	G	T	G	C	C	C	G	T	-	T	T	G	T
gag (433-452)	A	C	G	A	A	C	G	G	G	T	A	T	G	A	T	-
Strain	A6.BY.13.PV85															

U5 (103 - 122)	T	G	T	G	T	G	C	C	C	A	T	-	C	T	G	T
gag (433-452)	A	C	G	A	A	C	G	G	G	T	A	T	G	A	T	-
Strains	A1.CM.08.886_24, A6.UA.12.DEMA112UA024, B.DO.11.DEMB11DR001, B.PH.15.DEMB15PH003, 43_02G.SA.03.J11223, 49_cpx.GM.03.N26677															

U5 (103 - 122)	T	G	T	G	T	G	C	C	C	A	T	-	C	T	G	T
gag (433-452)	A	C	G	A	A	C	G	G	G	T	A	T	G	G	T	-
Strain	C.PK.14.DEMC14PK009															

U5 (103 - 122)	T	G	T	G	T	G	C	C	C	G	T	-	C	T	G	T
gag (433-452)	A	C	G	A	A	C	G	G	G	T	A	T	G	G	T	-
Strain	G.CD.03.LA23LiEd															

U5 (103 - 122)	T	G	T	G	T	G	C	C	C	G	T	-	C	T	G	T
gag (433-452)	A	C	G	A	A	C	G	G	G	T	G	T	G	A	T	-
Strain	J.CD.03.LA26DiAn															

U5 (103 - 122)	T	G	T	G	T	G	C	C	C	G	T	-	C	T	G	T
gag (433-452)	A	C	G	A	A	C	G	G	G	T	A	T	A	G	T	-
Strain	46_BF.BR.07.07BR_FPS625															

U5 (103 - 122)	A	G	T	C	C	A	C	T	G	G	G	-	C	G	A	T
gag (433-452)	A	C	G	A	A	C	G	G	G	T	A	T	G	A	T	-
Strain	A1.PK.15.PK034															

U5 (103 - 122)	A	A	T	A	A	A	G	C	C	G	A	-	A	A	A	T
gag (433-452)	A	C	G	A	A	C	G	G	G	T	A	T	G	A	T	-
Strain	02_AG.PK.15.PK032															

Supplementary Table 2. Mutational analysis showing phylogenetic base pair co-variation. [A] Conservation of U5-AUG structure and [B] Conservation of U5-*gag* putative interactions by comparison of all 178 HIV-1 Group M strains. Strains shown are those in which structure-conserving mutations were identified. Nucleotides in red represent nucleotides that were noted to participate in base pair semi co-variations (A-U or G-C to G-U or vice-versa). Mutations that do not conserve the predicted structures also exist in three of these strains (A1.PK.15.PK034 and 02_AG.PK.15.PK032 have mutations that disrupt U5-AUG and U5-gag, and 46_BF.BR.07.07BR_FPS625 has a single mutation that would disrupt the structure of U5-gag.)

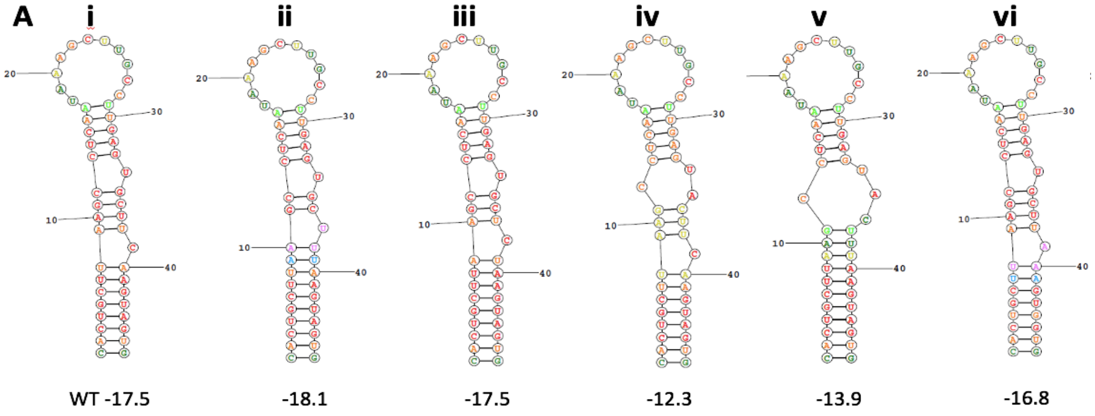

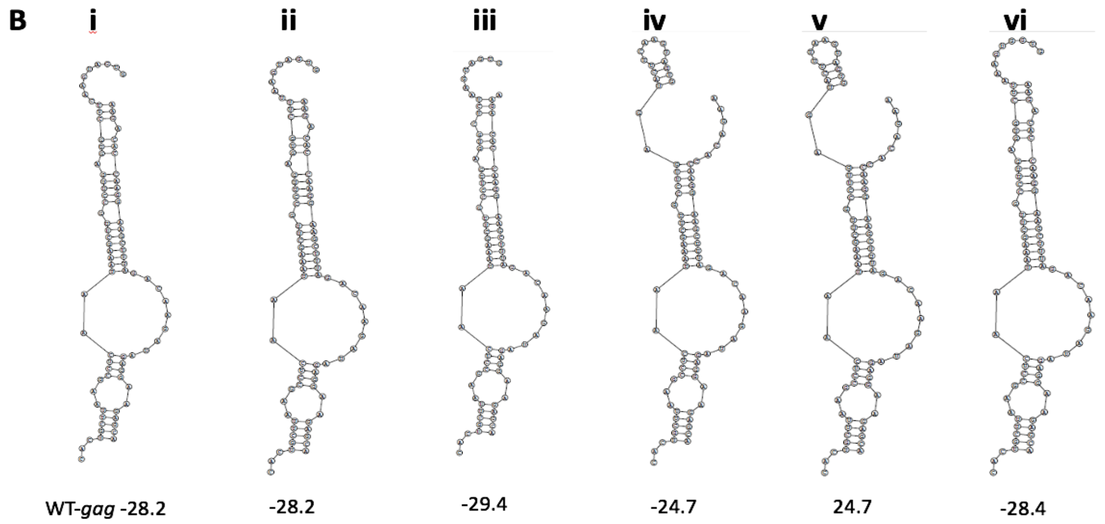

Supplementary Figure 1. [A] Predicted stability of the polyA structural motif amongst the frequently occurring polyA sequence variations of all M strains. [B] Predicted stability of the putative polyA-*gag* interaction amongst the frequently occurring polyA sequence variations of all M strains.

PolyA nucleotides 61-95 and *gag nucleotides* 622-660 (Figures 4 and 5) were interrogated for structural conservation. The depicted structures are derived from strain sequences containing the most frequently presented polyA mutantions in the Los Alamos database sequence compendium database 2018. i.B.FR.83.HXB2 WT strain, ii. A6.RU.11.11RU6950, 02_AG.KR.12.12MHR9 and 63_02A.RU.10.10RU6637 strains, iii. B.RU.11.11RU21n, C.MW.09.703010256_CH256.w96 and C.TZ.08.707010457_CH457.w8 strains, iv A1.CD.02.LA01AlPr, H.CD.04.LA19KoSa, and J.CD.03.LA26DiAn strains, v. F1.FR.04.LA22LeRe and H.CF.02.LA25LeMi strains, vi. 01_AE.PH.15.DE00115PH012 and 01_AE.TH.90.CM240 strains.

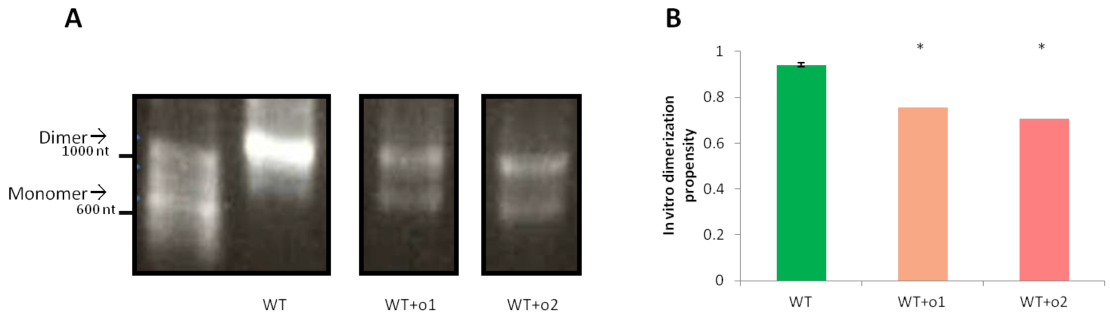

Supplementary Figure 2. Targeting of the U5-*gag* and polyA-*gag* structural interactions using antisense oligonucleotides [A] Representative TBM agarose gel from *in vitro* dimerization studies of the WT 690nt RNA fragment with/without antisense oligo nucleotides, which target the proposed polyA-*gag* (o1) and U5-*gag* (o2) putative interactions by binding to *gag* sequences. Separated lanes presented here show samples that were electrophoresed on the same gel. RNA size markers are shown. Image is representative of three independent replicates. [B] Average dimerization propensity ratio of the three independent replicates of the 690 nt long WT RNA fragment in the presence and absence of antisense oligos ($p<0.05$ for both WT+o1 and WT+o2). Error bars show standard deviation. Error bars are not visible on WT+o1 or WT+o2 due to low variance of the data.
